# Development and Pre-Clinical Analysis of Spatiotemporal-Aware Augmented Reality in Orthopedic Interventions

**DOI:** 10.1109/TMI.2020.3037013

**Published:** 2021-02-02

**Authors:** Javad Fotouhi, Arian Mehrfard, Tianyu Song, Alex Johnson, Greg Osgood, Mathias Unberath, Mehran Armand, Nassir Navab

**Affiliations:** Laboratory for Computational Sensing and Robotics, Johns Hopkins University, Baltimore, MD 21218 USA; Laboratory for Computational Sensing and Robotics, Johns Hopkins University, Baltimore, MD 21218 USA,; Laboratory for Computer-Aided Medical Procedures, Technical University of Munich, 80333 Munich, Germany; Laboratory for Computational Sensing and Robotics, Johns Hopkins University, Baltimore, MD 21218 USA; Department of Orthopaedic Surgery, Johns Hopkins Hospital, Baltimore, MD 21218 USA; Laboratory for Computational Sensing and Robotics, Johns Hopkins University, Baltimore, MD 21218 USA; Laboratory for Computational Sensing and Robotics, Johns Hopkins University, Baltimore, MD 21218 USA,; Department of Orthopaedic Surgery, Johns Hopkins Hospital, Baltimore, MD 21218 USA; Laboratory for Computational Sensing and Robotics, Johns Hopkins University, Baltimore, MD 21218 USA,; Laboratory for Computer-Aided Medical Procedures, Technical University of Munich, 80333 Munich, Germany

**Keywords:** Augmented reality, surgery, interaction, X-ray, frustum, visualization

## Abstract

Suboptimal interaction with patient data and challenges in mastering 3D anatomy based on ill-posed 2D interventional images are essential concerns in image-guided therapies. Augmented reality (AR) has been introduced in the operating rooms in the last decade; however, in image-guided interventions, it has often only been considered as a visualization device improving traditional workflows. As a consequence, the technology is gaining minimum maturity that it requires to redefine new procedures, user interfaces, and interactions. The main contribution of this paper is to reveal how exemplary workflows are redefined by taking full advantageof head-mounted displays when entirely co-registered with the imaging system at all times. The awareness of the system from the geometric and physical characteristics of X-ray imaging allows the exploration of different human-machine interfaces. Our system achieved an error of 4.76 ± 2.91 mm for placing K-wire in a fracture management procedure, and yielded errors of 1.57 ± 1.16° and 1.46 ± 1.00° in the abduction and anteversion angles, respectively, for total hip arthroplasty (THA). We compared the results with the outcomes from baseline standard operative and non-immersive AR procedures, which had yielded errors of [4.61mm, 4.76°, 4.77°] and [5.13 mm, 1.78°, 1.43°], respectively, for wire placement, and abduction and anteversion during THA. We hope that our holistic approach towards improving the interface of surgery not only augments the surgeon’s capabilities but also augments the surgical team’s experience in carrying out an effective intervention with reduced complications and provide novel approaches of documenting procedures for training purposes.

## I. Introduction

Interventional image guidance is widely adopted across multiple disciplines of minimally-invasive and percutaneous therapies [1]–[4]. Despite its importance in providing anatomy-level updates, visualization of images and interaction with the intra-operative data are inefficient, thus requiring extensive experience to properly associate the content of the image with the patient anatomy. These challenges become evident in interventions that require the surgeon to navigate wires and catheters through critical structures under excessive radiation, such as in fracture or endovascular repairs.

Surgical navigation and robotic systems are developed to support surgery with localization and execution of well-defined tasks [5]–[8]. Though these systems increase the accuracy, their complex setup and explicit tracking nature may overburden the surgical workflow and consequently impede their acceptance in clinical routines [9]. Image-based navigation alleviates the requirements for external tracking, though depends strongly on pre-operative data which become outdated when the anatomy is altered during the surgery [10], [11].

As the expectations from surgical outcomes increase with more advanced technologies introduced in the ORs, the communication between the surgeon, crew, and the information becomes an important concern. Ineffective communication leads to increase of surgery time, radiation, and frustration to a point where in fluoroscopy-guided procedures, instead of the X-ray technician, the surgeons may reposition the scanners to ensure the task-defined views are optimal [12], [13].

To bridge the inefficiency gaps in surgical workflows, researchers have investigated the importance of human factor considerations in improving the usability of surgical data [14]–[16]. Recent works focused on facilitating the unmet interaction needs by introducing touch-less mechanisms such as gaze, foot, or voice commands [17], [18]. We believe the high stakes of surgery necessitates efficient interaction between all actors in the operating room *i*.*e*. surgeon, anesthesiologist and staff to communicate and access information. This demands user-centric designs that can also accommodate fluid movement of information and surgical inference across the entire team.

Augmented reality (AR) solutions have gained popularity in computer-integrated surgeries, as they can provide intuitive visualizations of medical data directly at patients’ site. Early works on surgical AR focused largely on multi-modal fusion of information and provided display-based overlays [19]–[21]. Subsequently, AR enabled the utilization of pre- and intra-interventional 3D data during therapies [22]–[25].

The emergence of commercially available optical see-through head-mounted display (HMD) systems has led to development of AR solutions for various image-guided surgical disciplines, including percutaneous vertebroplasty, kyphoplasty, lumbar facet joint injection, orthopedic fracture management, bone cancer treatment, total hip arthroplasty (THA), interlocking nailing, cardiovascular surgeries, and surgical education [16], [26]–[33].

HMD-based AR has served as image viewer that directly displays the data at the operative site using virtual fluoroscopy monitors, hence eliminating the conventional off-axis visualization through static monitors [34]. Moreover, AR is used to provide navigational information during interventions [35]–[37]. These systems often rely on tracking of external markers, which require line-of-sight and invasive implantation into patients tissue that can hinder their usability. Andress *et al*. suggested a flexible marker-based surgical AR methodology which only required the marker to appear in the X-ray beam during the image acquisition, and was removed immediately after [38]. Recent inside-out localization strategies in AR have greatly favored the fluid workflow over explicit navigation, and have proved effective in eliminating the need for external markers [39]–[41].

**Fig. 1.**
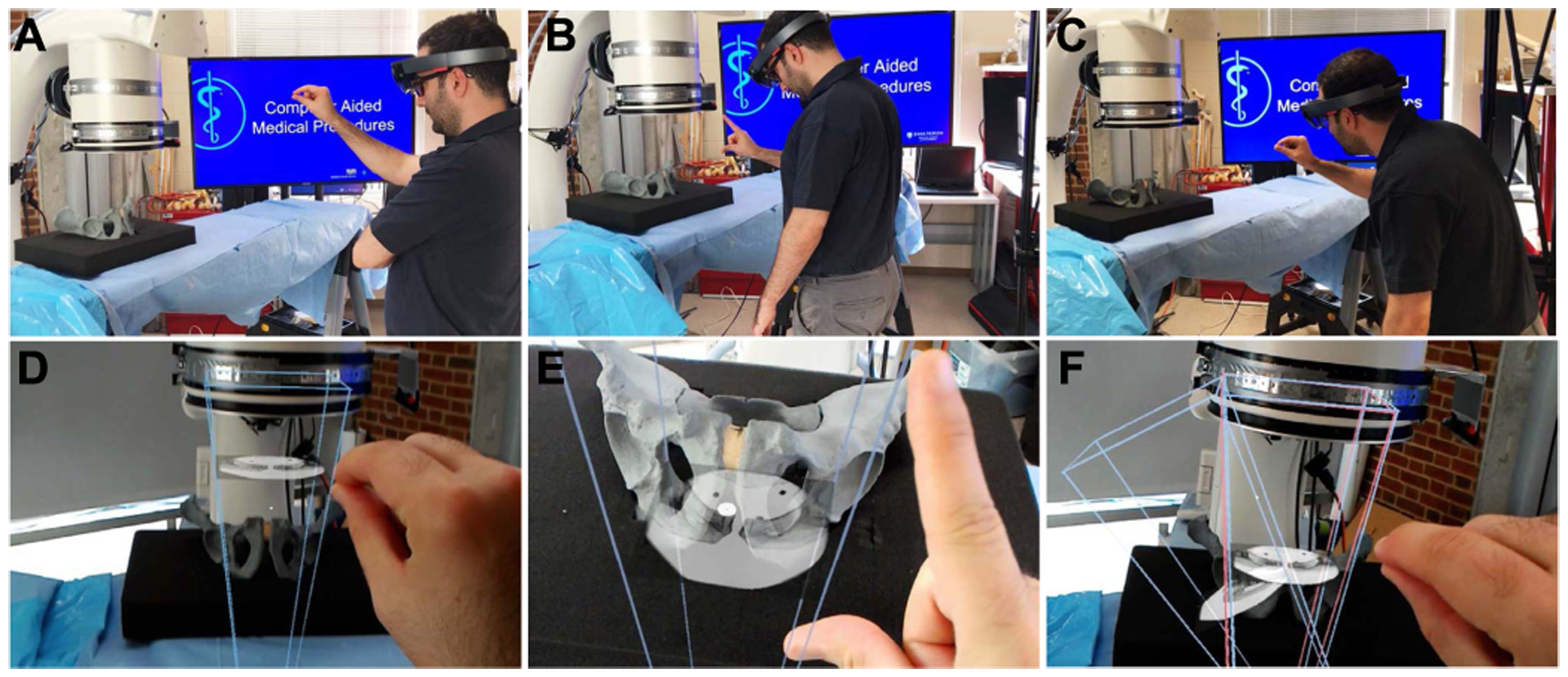
Spatiotemporal-aware AR exploits the full imaging geometry. The augmented user interacts with the X-ray images within their viewing frustums (**A-C**). Corresponding AR views are shown in **D-F**.

This manuscript introduces the methodology and usability of a novel spatially-aware concept that enables immediate interaction with the medical data and promotes team approach where all stake-holders share a unified AR experience and communicate effectively. Our methodology exploits the viewing frustum of the imaging devices and human observers in the operating room (Fig. 1), and provides an engaging and immersive experience for the surgical team. We built upon the concept of image frustums [41], and designed the complete methodology that integrates spatiotemporal-aware AR in the entire workflow. Our contributions are particularly centered around intra-operative planning, integration of the planning into the surgical workflow, and pre-clinical analysis. We showcase this solution in two high volume orthopedic procedures, *i*.*e*. K-wire placement in fracture care surgery, and acetabular cup placement in THA.

## II. Methodology

Our main contributions are the collaborative AR concepts using spatiotemporal-aware flying frustums [41] that enable intra-operative planning, define new workflows, support surgical crew, enhance the communication between surgeon and data, and enable intuitive documentation of the surgery for training purposes. We present the methodology for the realization of these concepts in the remainder of this section.

### A. Spatial-Awareness for AR

Visual data from cameras contain a wealth of information that can be used for simultaneous localization and mapping (SLAM). Visual SLAM is an important ingredient in our AR-based interaction recipe that enables co-localization of augmented users and the imaging device, which we will refer to as imaging observer, in a shared operating room environment. Marker-free co-localization will enable the 3D information to easily propagate through different bodies and be spatially-registered for all HMD users.

In the first step, each AR user is localized within the environment. The relative pose between two frames *α* and *β* using the environment map **M** can be estimated by minimizing the following reprojection function:

(1) $${}^{\alpha }T_{\beta }=\underset{\alpha {\hat{T}}_{\beta }}{\text{arg}\;\text{min}}\;D\left({}^{\alpha }{\hat{T}}_{\beta },M\right)\;=\underset{\alpha {\hat{T}}_{\beta }}{\text{arg}\;\text{min}}\underset{f_{i}^{\left(\alpha \right)}\in I\alpha }{\sum }{\left|f_{i}^{\left(\alpha \right)}-P\left(M\left(f_{i}^{\left(\alpha \right)}\right)\right)\right|}^{2}\;+\underset{f_{i}^{\left(\beta \right)}\in I\beta }{\sum }{\left|f_{i}^{\left(\beta \right)}-P^{\alpha }{\hat{T}}_{\beta }\left(M\left(f_{i}^{\left(\beta \right)}\right)\right)\right|}^{2},$$

where $f_{i}^{\left(\alpha \right)}$ and $f_{i}^{\left(\beta \right)}$ are corresponding features in images *I*_*α*_ and *I*_*β*_, and *P* is the projection operator. In direct SLAM, the features include all pixels, and in indirect SLAM, the features are a sparse set of keypoints in the image. In Eq. 1, we optimize for the 6 parameters of a rigid transformation that best explains the pose between *α* and *β* using only the features present in the images. This step, also known as environment tracking, has become an standard part of most AR applications.

**Fig. 2.**
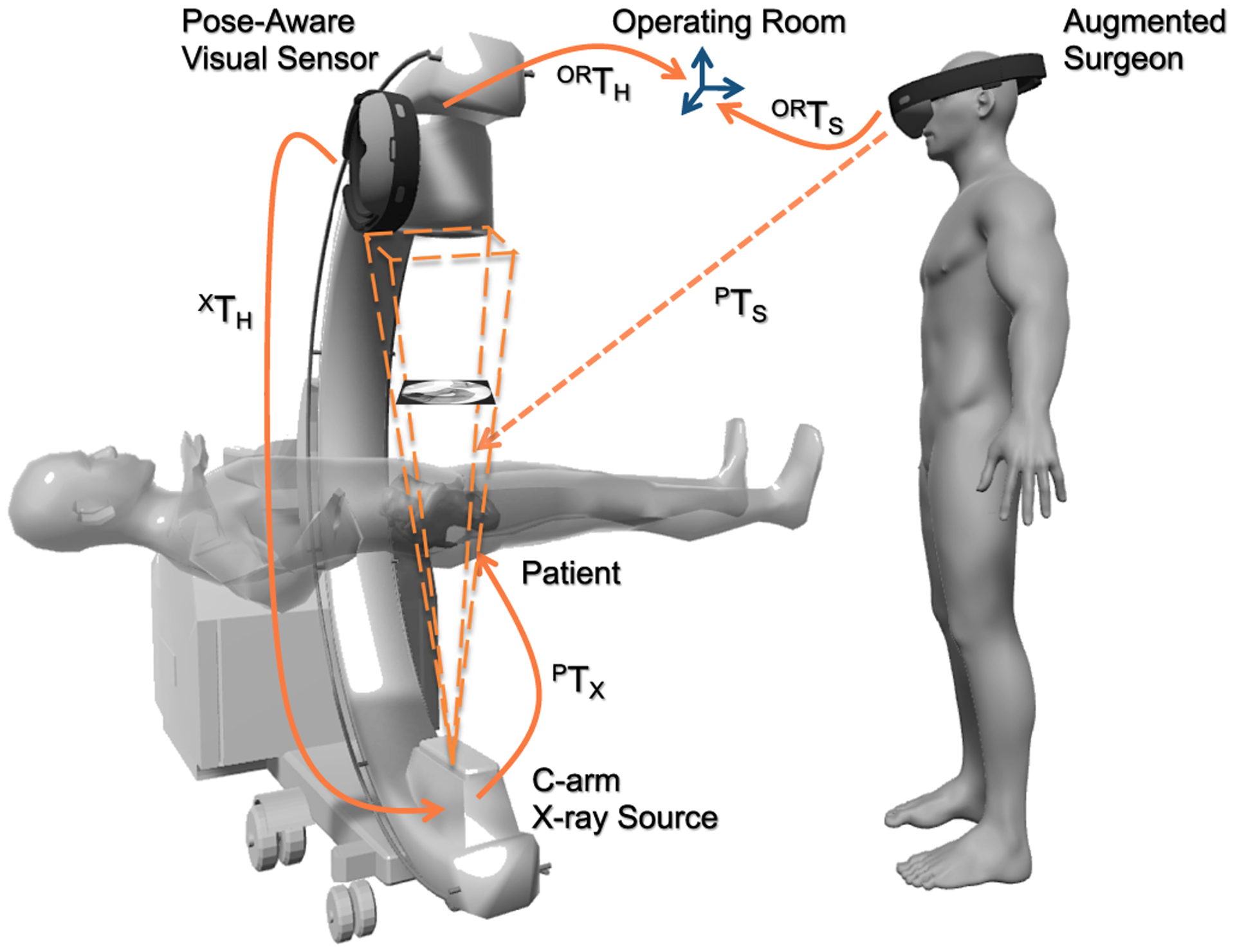
The transformation chain of the spatially-aware AR system is shown for a C-arm fluoroscopy system. The transformations layout show the closed-loop between the imaging device and the users at all time. The same relations can be generalized to include multiple users.

In a similar fashion, all users can be localized with respect to the first user, or with respect to a common spatial anchor in the operating room. The first member joining the shared experience will establish the anchor, *i*.*e*. OR coordinate system, and every other member of the AR session will share their pose in a master-slave configuration with respect to this OR frame [39]. This relation is shown as ^OR^**T**_S_ in Fig. 2.

### B. Imaging Observer

C-arm scanners offer fluorscopic imaging capabilities for a wide range of less-invasive therapeutic areas. To seamlessly integrate this imaging device into our interactive AR paradigm, we augment the scanner with a rigidly attached visual tracker, that observes the structures in the OR environment, and communicates spatial information to all users. The materialization of this imaging observer system requires a co-calibration between the visual tracker on the scanner, and the X-ray source [40]. The constant transformation that explains the calibration is denoted as ^X^**T**_H_ in Fig. 2.

To estimate ^X^**T**_H_, we formulate an over-determined system of equations as follows:

(2) $${}^{\text{IR}}T_{\text{OR}}{=}^{\text{IR}}T_{\text{X}}{\left(t_{i}\right)}^{\text{X}}T_{\text{H}}{}^{\text{OR}}T_{\text{H}}^{-1}\left(t_{i}\right)\;{=}^{\text{IR}}T_{\text{X}}{\left(t_{i+1}\right)}^{\text{X}}T_{\text{H}}{}^{\text{OR}}T_{\text{H}}^{-1}\left(t_{i+1}\right).$$

IR denotes the frame of an external tracker that is used to track the motion of the C-arm source as it undergoes different motion at times *t*_*i*_ and *t*_*i*+1_. To construct Eq. 2, the scanner is oriented in different poses, at each of which ^IR^**T**_X_ and ^OR^**T**_H_, which are the poses of the X-ray source in the external tracker frame and the SLAM-based localization of the HMD in the OR coordinate system, respectively, are recorded. It is important to note that the IR tracker is only used for this one-time and offline co-calibration, and it is not used intra-operatively. By re-arranging Eq. 2, we formulate the problem in the form of **AX** = **XB** as presented in Eq. 3, such that **X** ≔^X^ **T**_H_, and **A** and **B** represent the relative motion of the X-ray source and the SLAM capable visual sensor on the gantry, respectively.

(3) $${}^{\text{IR}}T_{\text{X}}^{-1}{\left(t_{i+1}\right)}^{\text{IR}}T_{\text{X}}{\left(t_{i}\right)}^{\text{X}}T_{\text{H}}{=}^{\text{X}}T_{\text{H}}{}^{\text{OR}}T_{\text{H}}^{-1}{\left(t_{i-1}\right)}^{\text{IR}}T_{\text{X}}^{-1}\left(t_{i}\right).$$

Rotation and translation components of the hand-eye problem are disentangled and computed separately as:

(4) $$R_{\text{A}}R_{X}=R_{X}R_{B}\;R_{\text{A}}{\text{t}}_{X}+t_{A}=R_{X}t_{B}+t_{X}.$$

We estimate the rotation parameters using unit quaternion representation as *q*_**A**_ *q*_**X**_ = *q*_**X**_ *q*_**B**_. Given that a unit quaternion *q*_**i**_ is formed by a vector **v**_**i**_ and a scalar *s*_**i**_ such that *q*_**X**_ = **v**_**X**_ + *s*_**X**_, we re-write the rotation component in Eq. 4 using the quaternion product rule as:

(5) $$\vec{\left(.\right)}:s_{A}v_{X}+s_{X}v_{A}+v_{A}\times v_{X}=s_{X}v_{B}+s_{B}v_{X}+v_{X}\times v_{B}\;\text{ (}\;\text{.) : }s_{A}s_{X}-v_{A}\cdot v_{X}=s_{X}s_{B}-v_{X}\cdot v_{B}.$$

Re-arranging the above formulation yields:

(6) $$\left[\begin{array}{cc} s_{A}-s_{B} & {\left(v_{A}-v_{B}\right)}^{T}\\ \left(v_{A}-v_{B}\right) & \left(s_{A}-s_{B}\right)I_{3}+{\left[v_{A}+v_{B}\right]}_{\times } \end{array}\right]\left[\begin{array}{l} s_{X}\\ v_{X} \end{array}\right]=\left[\begin{array}{c} 0\\ 0_{3} \end{array}\right],$$

which is then solved in a constrained optimization fashion as:

(7) $$\text{min}∥Mq{∥}_{2}^{2}\;\text{ s}\;\text{.t}\;\text{. }\;\;∥q{∥}_{2}^{2}=1,$$

where $q={\left[s_{X},{\vec{v}}_{x}\right]}^{T}$. After the rotation parameters are computed, the translation vector is estimated in a least-squares setting: (*R*_**A**_ − *I*_3_)**t**_**X**_ = *R*_**X**_**t**_**B**_ − **t**_**A**_.

At the end of this phase, we have achieved *R*_X_ and **t**_X_ that express the calibration between the X-ray source and the integrated visual tracker; hence, we directly access the pose of the X-ray camera from the pose information acquired from the visual tracker on the scanner.

### C. Geometry-Awareness for AR

In this section, we describe the underlying geometry that allows us to combine the content of 2D X-ray images, directly with the 3D spatial information we computed in Sec. II-A and II-B. To this end, we explicitly model the viewable region of the X-ray camera, known as the flying frustum [41], and allow interaction with images within their geometries. It is important to note that, the flying frustum refers to the full pyramid of vision (Fig. 2), and is different than the truncated pyramids used in the computer graphics community. Despite the similarities in formulation, the conventional frustum model in graphics only applies to reflective images, and cannot accommodate the transmission model used in fluoroscopy. Therefore, we extend the perspective pinhole camera model that is commonly used in the computer vision community [42].

In our paradigm, users can move the images within their frustums on a virtual plane known as the *near* plane, between X-ray source and detector (referred to as the *far* plane), while they remain a valid image of the same anatomy. This interaction enables the users to intersect the images with corresponding anatomies, and intuitively observe *2D-image-to-3D-anatomy* associations. Additionally, the imaging technologists which operate the scanner, can align the scanner with a desired frustum that is decided by the surgeon.

A flying frustum is defined using the following model:

(8) $$P_{f}=\left[\begin{array}{ccc} \frac{n}{f} & 0 & 0\\ 0 & \frac{n}{f} & 0\\ 0 & 0 & 1 \end{array}\right]K\;P\left[\begin{array}{cc} {}^{\text{OR}}R_{\text{X}} & {}^{\text{OR}}t_{\text{X}}\\ 0^{⊤} & 1 \end{array}\right],$$

where *n* refers to the distance to the *near* plane, *f* is the focal length, *K* is the matrix of intrinsic parameters, and 0 ≤ *n* ≤ *f*. The parameter *n* is controlled by the user, such that when *n* = *f*, the X-ray image is directly displayed at the detector scale. It is worth mentioning that, with conventional frustum models, the *near* plane can only take values smaller than the *far* plane, which is not the case in our representation.

For each 2D point $x_{i}\in I$, where I is the domain of all acquired images, the corresponding point *x_f_* in the frustum domain F is scaled by a factor *s* as $x_{f}=sx_{i}=\left(\frac{n}{f}\right)x_{i}$, such that 0 ≤ *n* ≤ *f*. Finally, the 3D pose of the interactive image in the frustum is defined as:

(9) $${}^{\text{OR}}T_{\text{I}}=\left[\begin{array}{cc} {}^{\text{OR}}R_{\text{X}} & {}^{\text{OR}}t_{\text{X}}\\ 0^{⊤} & 1 \end{array}\right]\left[\begin{array}{cc} & 0\\ I_{3} & 0\\ & n\\ 0^{⊤} & 1 \end{array}\right]\;=\left[\begin{array}{ll} & r_{13}n\\ {}^{\text{OR}}R_{\text{X}} & r_{23}n{+}^{\text{OR}}t_{\text{X}}\\ & r_{33}n\\ 0^{⊤} & 1 \end{array}\right],$$

where $R={\left\{r_{i,j}\right\}}_{i,j:1,2,3}.$

### D. Planning Using Flying Frustums

**Fig. 3.**
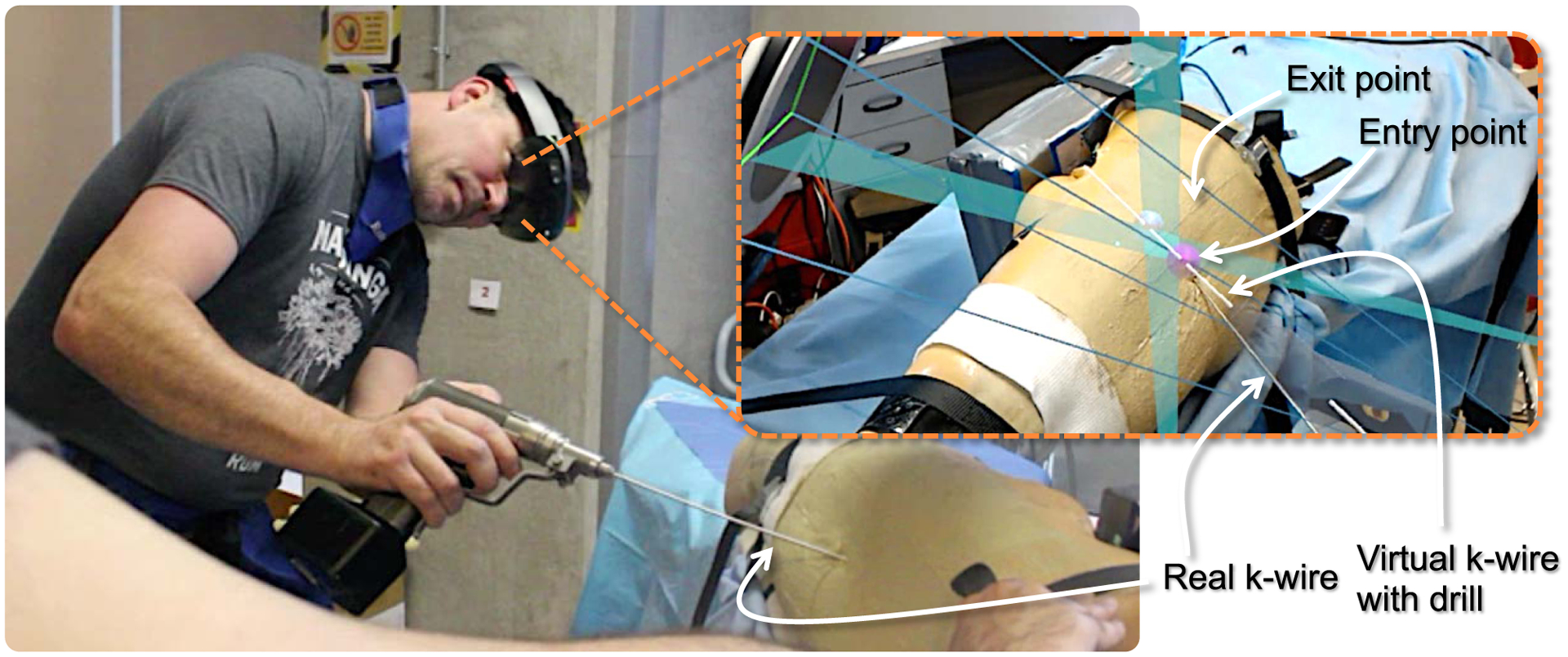
In K-wire placement for fracture reduction procedures, the surgeon can plan the entry and exit points of the wire on two X-ray images. After the planning, two triangular planes are constructed by connecting the drilling trajectory defined on the detector plane (X-ray image) and the C-arm source (X-ray origin). The intersection of these two planes is a line that corresponds to the desired drilling trajectory in 3D. By exploiting the imaging frustum, this line is augmented directly on the patient anatomy. The surgeon can then align the physical drill with its virtual counterpart, and advance the wire through the anatomy.

Flying frustums discussed in Sec. II-A–II-C embed sufficient 3D and 2D information that enable interventional planning for the placement of surgical tools (Fig. 3). In this section we introduce two distinct approaches for intra-operative planning. In the first planning approach, we forward-project the 3D virtual surgical implants onto the X-ray images within each frustum using the respective X-ray projective geometry. Hence, the user can observe the implant’s projection in the same X-ray image and verify the implant’s appearance before placing the real implant. This approach is generic and allows the planning of implants with any arbitrary shape. In contrast to the earlier works that overlay on the X-ray image the surgical tools that are tracked using navigation systems [43], our methodology enables intra-operative planning by projecting and moving a virtual implant (not a physically tracked implant) until it aligns with target structures in all valid frustums and X-ray images.

In the second approach, we use multi-view geometry to reconstruct landmark targets or trajectories in 3D. In both methods, after the respective planning on the flying frustums, the resulting 3D information, already registered to the anatomy, is visualized on the patient. In the following, we describe each of the two approaches.

**Fig. 4.**
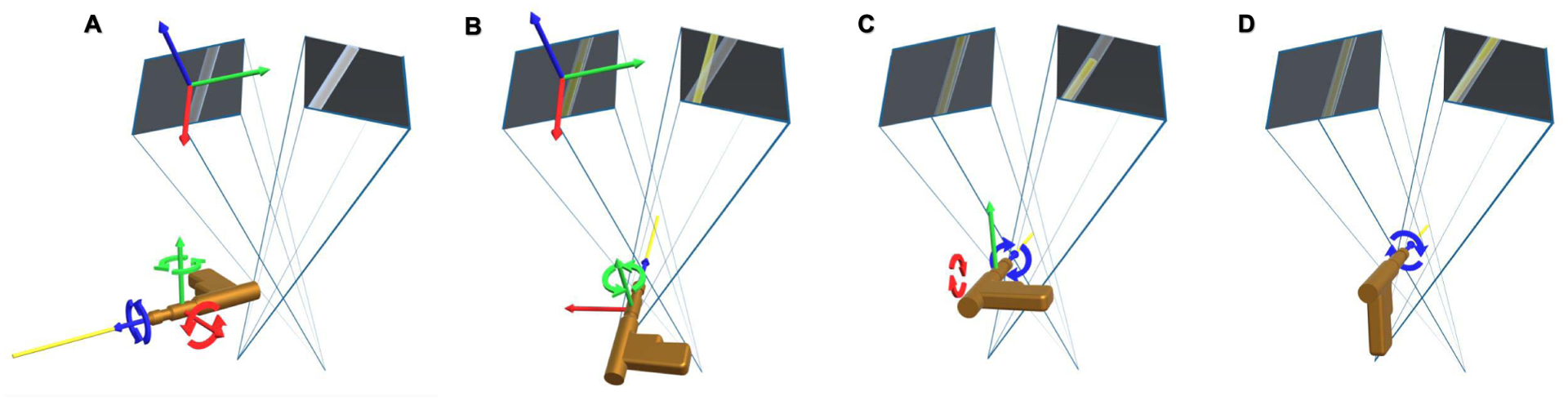
The coordinate frame of the virtual drill is defined as shown in **A**, where the *Z*-axis that is shown in blue points out of the drill along with the K-wire. The corresponding *X*- and *Y*-axes are shown in red and green, respectively. **B** shows the manipulation of the virtual drill with 4 DOF until the projection of the K-wire is aligned in the first frustum (the yellow projection of the wire is contained within the tube in the left frustum). These 4 DOFs are sufficient to align the tool appropriately with the target anatomy in the first image. In the next step, we change the transformation constraints, as shown in **C**; after the alignment of the drill with the anatomy is verified in the first frustum, the drill maintains rotational freedom around its local *X*- and *Z*-axes, and translational freedom along its *Y*- and *Z*-axes. These DOF constraints allow the implant’s alignment in the second frustum while maintaining the alignment between tool and anatomy in the first frustum. Finally, **D** shows the virtual drill being restricted to only 2 DOF. Moving and rotating along these two DOFs will not influence the alignment in either of the two frustums.

In the first method, virtual tools are manipulated in 3D by the user, and simultaneously projected onto the X-ray images of all valid frustums. A point $X_{t}\in T$, where T is the domain of all 3D points on a virtual tool, is projected onto the *i*^th^ frustum as $x_{t_{i}}=P_{f_{i}}X_{t}$. The virtual tool is manipulated with complete 6 *degrees-of-freedom* (DOF) by the user to plan on all the frustums simultaneously. However, it may appear challenging to align concurrently in both views. Alternatively, we can apply rotational and translational constraints to the virtual tool, such that the tool can be aligned first in one frustum, and then in the second frustum, while the alignment in the first one is preserved. As shown in Fig. 4, in the first stage, the *Y* -axis of the virtual tool is rotated to hold the same direction as the *Z*-axis of the first frustum. The 4 DOF transformation model of the virtual tool is defined as:

(10) $$T^{f_{1}}=\left[\begin{array}{cccc} \text{cos}\theta & 0 & \text{sin}\theta & t_{x}\\ 0 & 1 & 0 & t_{y}\\ -\text{sin}\theta & 0 & \text{cos}\theta & t_{z}\\ 0 & 0 & 0 & 1 \end{array}\right],$$

where *θ* is the rotation angle around the local *Y* -axis and *t*_*x*_, *t*_*y*_ and *t*_*z*_ are translations along *X*, *Y* and *Z*-axes, respectively. In the second stage, the virtual tool is locked to only allow rotation around the *X* and *Z*-axes, and translation in *Y* and *Z*-axes using the transformation model as:

(11) $$T^{f_{2}}=\left[\begin{array}{cccc} \text{cos}\psi & -\text{sin}\psi \text{cos}\phi & -\text{sin}\psi \text{sin}\phi & 0\\ \text{sin}\psi & \text{cos}\psi \text{cos}\phi & -\text{cos}\psi \text{sin}\phi & t_{y}\\ 0 & \text{sin}\phi & \text{cos}\phi & t_{z}\\ 0 & 0 & 0 & 1 \end{array}\right],$$

where *ϕ* and *ψ* are the rotation angles around their *X* and *Z*-axes, respectively. As shown in Fig. 4, **T**^*f*2^ does not influence the alignment of the tool and target in the image of the first frustum. Finally, after alignment in both frustums is achieved, the virtual tool then will only be constrained to have 2 DOF which is defined as:

(12) $$T^{\text{tool}}=\left[\begin{array}{cccc} \text{cos}\psi & -\text{sin}\psi & 0 & 0\\ \text{sin}\psi & \text{cos}\psi & 0 & 0\\ 0 & 0 & 1 & t_{z}\\ 0 & 0 & 0 & 1 \end{array}\right].$$

**Fig. 5.**
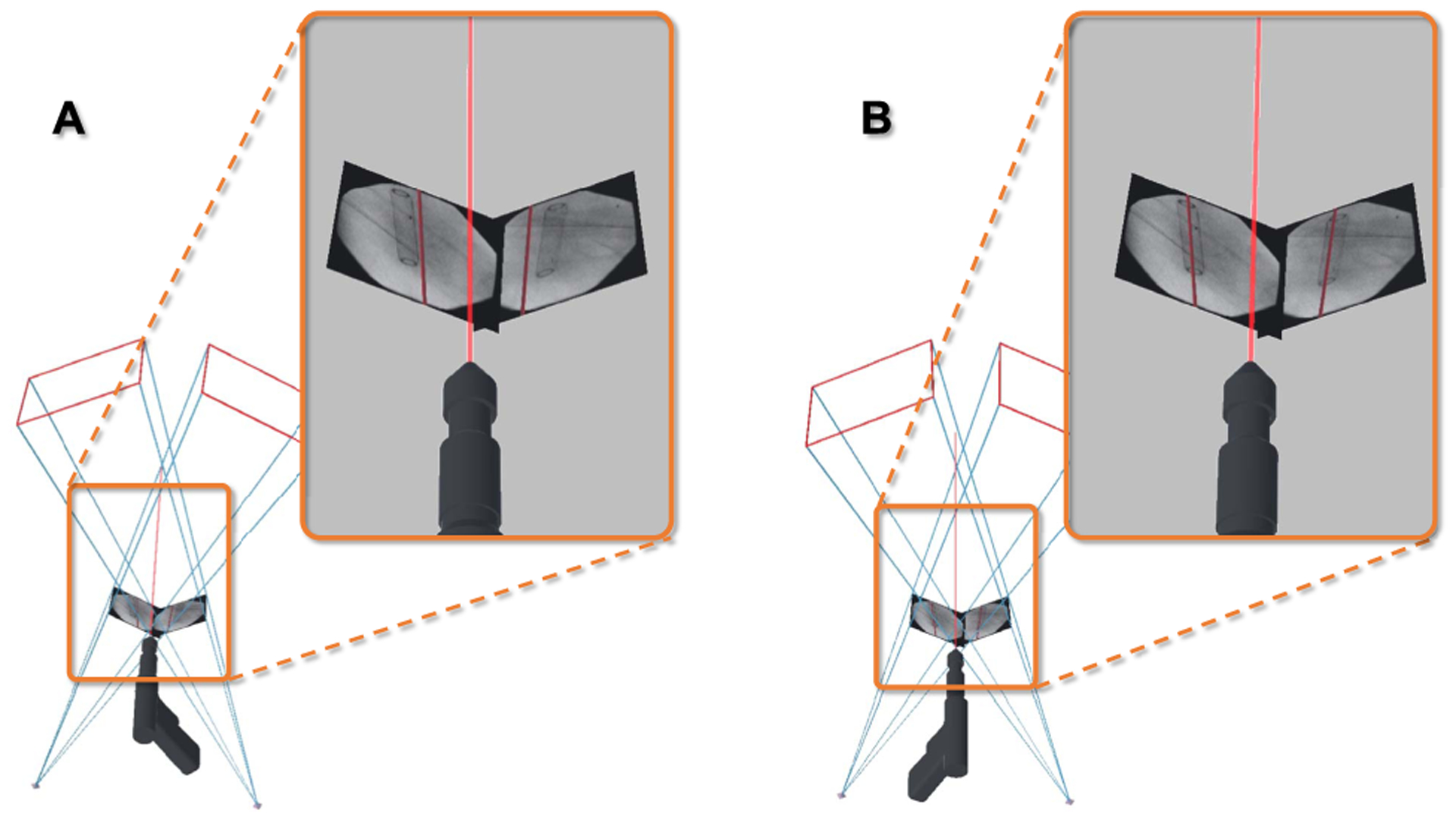
The augmented projections allow us to exploit the geometry in AR and plan surgical tools in relation to patient anatomy. The misaligned virtual drill in **A** is repositioned until it appears inside the desired structure in all the frustums (**B**).

In an exemplary case shown in Fig. 5, the virtual drill is rotated and translated until it passes through a desired structure (e.g. through a bone canal) in all frustums. An alignment consensus in all frustums is the equivalent of the alignment of the virtual 3D tool with the imaged anatomy in 3D.

**Fig. 6.**
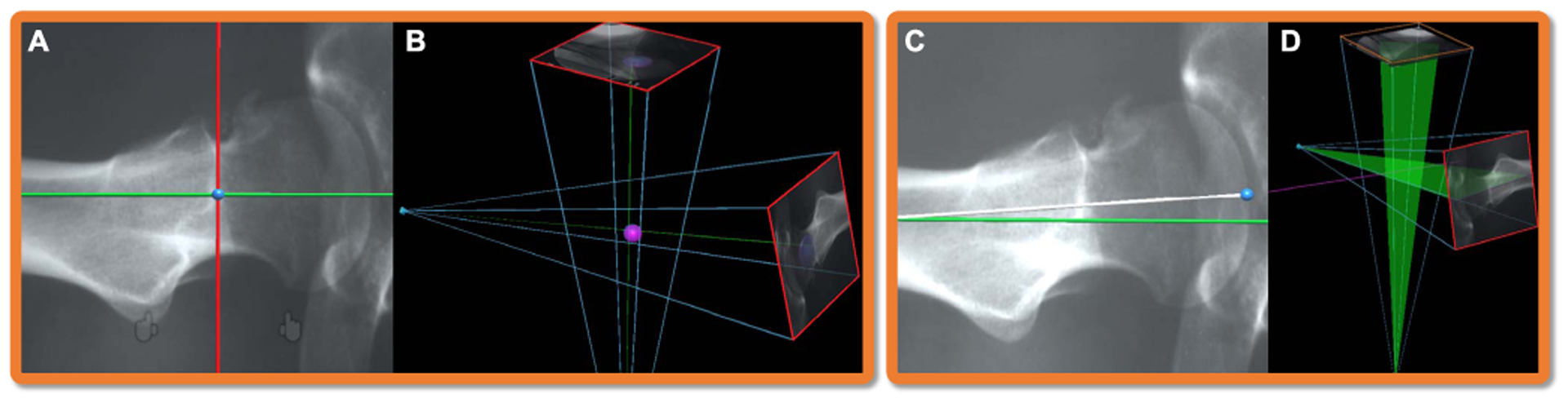
Each point in a frustum image corresponds to a ray passing through the landmark in 3D, and connecting the source and detector of the C-arm. Intersection of two rays recovers the 3D point and renders it directly on the patient (**A-B**). Similarly, annotation of lines in each frustum, corresponds to a plane in 3D. The intersection of these planes restores the 3D planning trajectory, and renders it in AR such that it travels through the corresponding anatomical structure (**C-D**).

The second planning approach requires 2D interaction on the frustum X-ray images. In this setting, for each selected landmark on a frustum image, a 3D ray connecting the C-arm source and the target landmark will be rendered into the AR scene. As illustrated in Fig. 6, the intersection of two rays from a corresponding landmark in two images reconstruct the 3D landmark. Each ray is defined via two elements: *i*) the position of the C-arm X-ray source **c**_*i*_, and *ii*) the unit direction vector **u**_*i*_ from the source to the annotated landmark in the frustum. We estimate the closest point $x_{l}^{*}$ to the *N* = 2 rays corresponding to each landmark *l* via a least-squares minimization strategy as follows:

(13) $$x_{l}^{*}=\underset{x\in {\mathbb{R}}^{3}}{\text{arg}\;\text{min}}\overset{N}{\underset{i=1}{\sum }}∥\left(I_{3}-u_{i}u_{i}^{⊤}\right)x-t_{i}{∥}^{2},\;\text{ where }t_{i}=\left(I_{3}-u_{i}u_{i}^{⊤}\right)c_{i}.$$

Similarly, two points on a frustum *i* defining the entry and the exit points of a drilling trajectory, associate to two rays **u**_1*i*_ and **u**_2*i*_ in 3D. These two rays span a plane in 3D as shown in Fig. 6. The intersection of the planes corresponding to the same entry and exit points on frustums *i* and *j* form a 3D line **d**_12_ = (**u**_1*i*_ × **u**_2*i*_) × (**u**_1*j*_ × **u**_2*j*_) that passes through the desired entry and exit points on the patient anatomy.

Our first approach requires a more complex interaction with the augmented surgical implant using the 6 *degrees-of-freedom*, however generalizes to arbitrary structures beyond linear annotations, such as the curved plates used for internal fixations. In our second approach, if the 3D model of the implant is known, the planning can be achieved by annotating only three corresponding landmarks in each frustum image.

To perform intra-operative planning with flying frustums, two X-ray images are sufficient. During these two acquisitions, we assumed that the patient is static. For many orthopedic and trauma interventions, particularly for K-wire placement, this is an entirely valid assumption.

### E. Surgical Workflow Integration

**Fig. 7.**
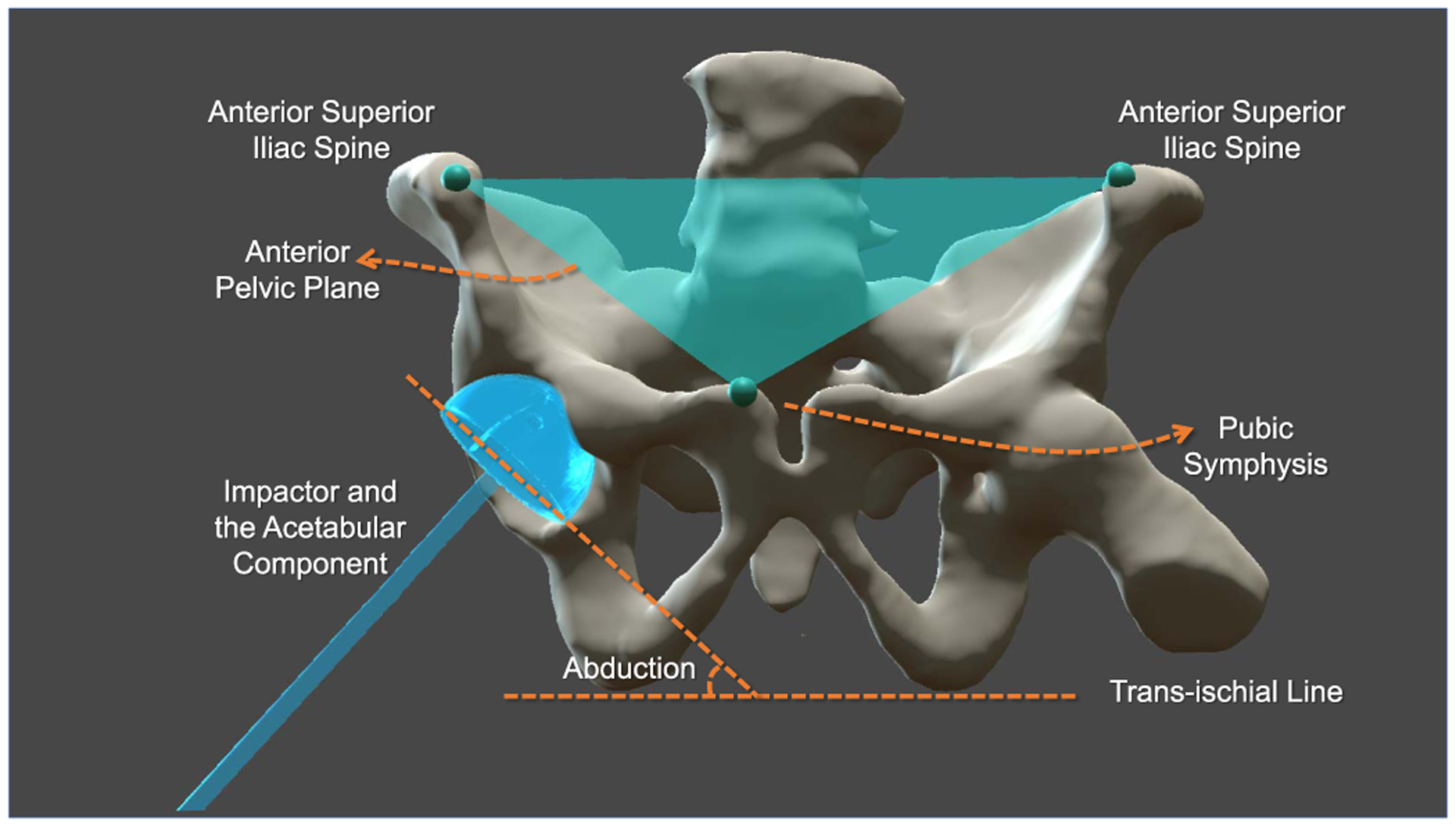
In THA, abduction and anteversion angles of the acetabular implant are defined with respect to the anterior pelvic plane (APP). Abduction refers to the in-plane rotation, and anteversion refers to the out-of-plane rotation of the cup. The anterior pelvic plane is defined based on three points: the left and right anterior superior iliac spine landmarks, and the pubic symphysis. Once the surgeon annotates these landmarks, we identify the APP, and subsequently, render the acetabular components at appropriate angles. We also let the user annotate the center of the acetabulum in two or more views, which is used to calculate the 3D position of this landmark on the patient, hence allowing the center of the hemispheric component to render inside the hip socket. In the execution phase, the user aligns the real impactor and cup with their virtual counterparts.

Intra-operative planning and execution with the flying frustums support can be used in various fluoroscopy-guided procedures. In THA, the critical points defining the anterior pelvic plane (APP) can be each identified on X-ray images. These anatomical landmarks include the left and right anterior superior iliac spine points on the pelvic wing and the pubic symphysis. Given APP, a virtual acetabular implant and a rigidly attached impactor are rendered in AR with their desired orientation that is calculated with respect to APP. Likewise, the translational component of the cup implant is identified by defining the center of the patient acetabulum on corresponding fluoroscopic images. These relations are shown in Fig. 7. Once these intra-operative planning steps are completed by the surgeon, virtual representations of the cup and impactor are augmented over the patient’s acetabulum with the appropriate abduction and anteversion angles. The surgeon could then align the impactor with its virtual counterpart reducing the amount of required X-rays. It is important to note that achieving the desired angles for the hip implant is a crucial step that is mentally challenging to verify from single view X-ray images, as it is commonly practiced in the direct anterior approach.

**Fig. 8.**
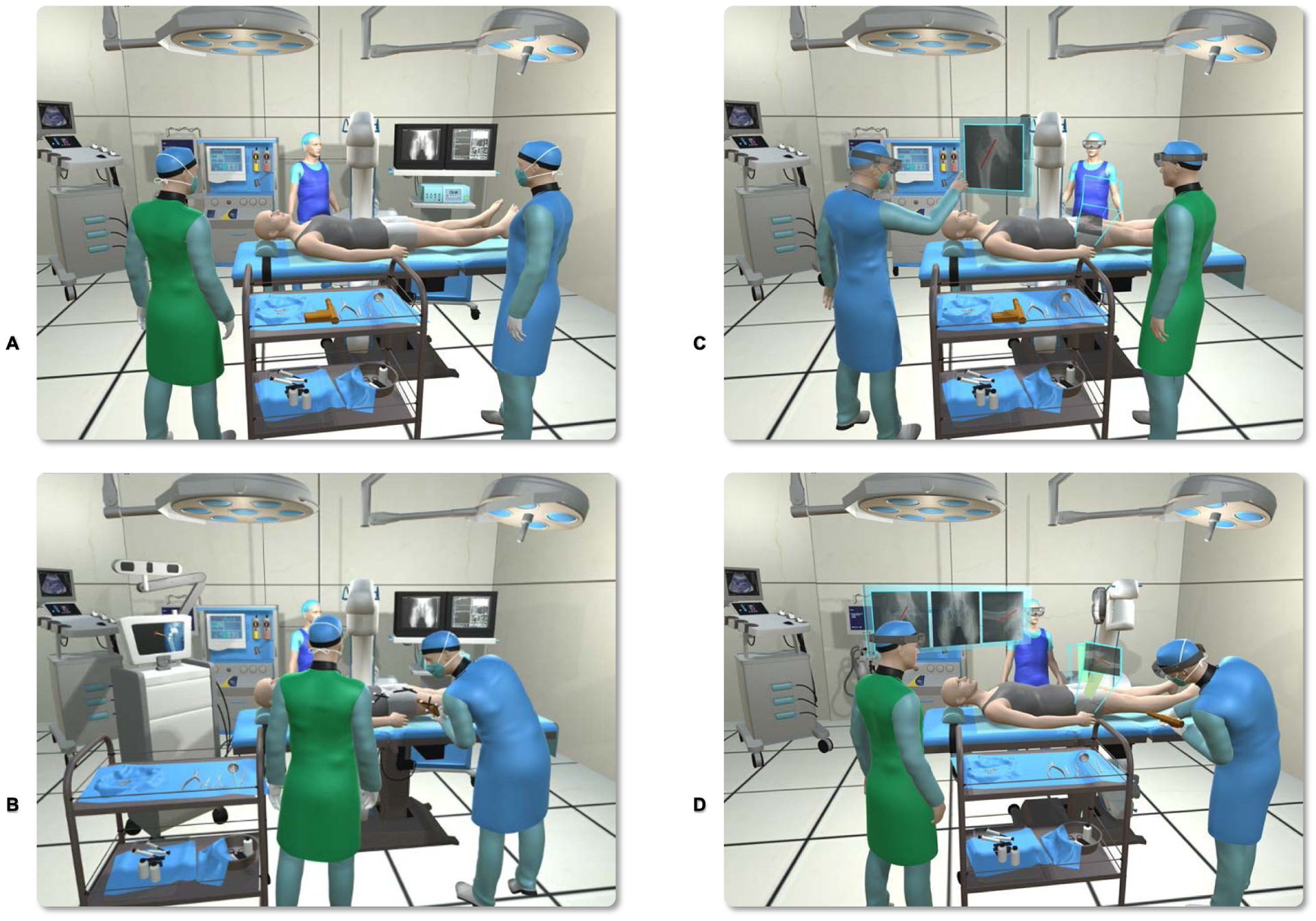
The standard operative procedure in percutaneous orthopedic interventions makes extensive use of interventional imaging **(A)**. Classic navigation-based solutions use sophisticated tracking hardware and external markers to provide geometric registration between the content in the image and the patient **(B)**. On the other hand, in the AR-enhanced OR that we suggest **(C)**, the surgeon and crew interactively use the data and pass the information around without explicit navigation. Based on the concepts introduced in Sec. II-D, the planning on X-ray images is directly visualized on the patient. The surgeon takes action based on the information from planning, as well as the X-ray images that are positioned within their respective frustums, both of which are seen through the HMD **(D)**.

Another exemplary image-guided procedure is the placement of screws and K-wires during fracture management. As shown in Fig. 8, AR provides support for placement of K-wires using the trajectory planning on the corresponding frustums. Fig. 8 also depicts the use of our AR solution in the OR, and compares the proposed environment with the current OR.

**Fig. 9.**
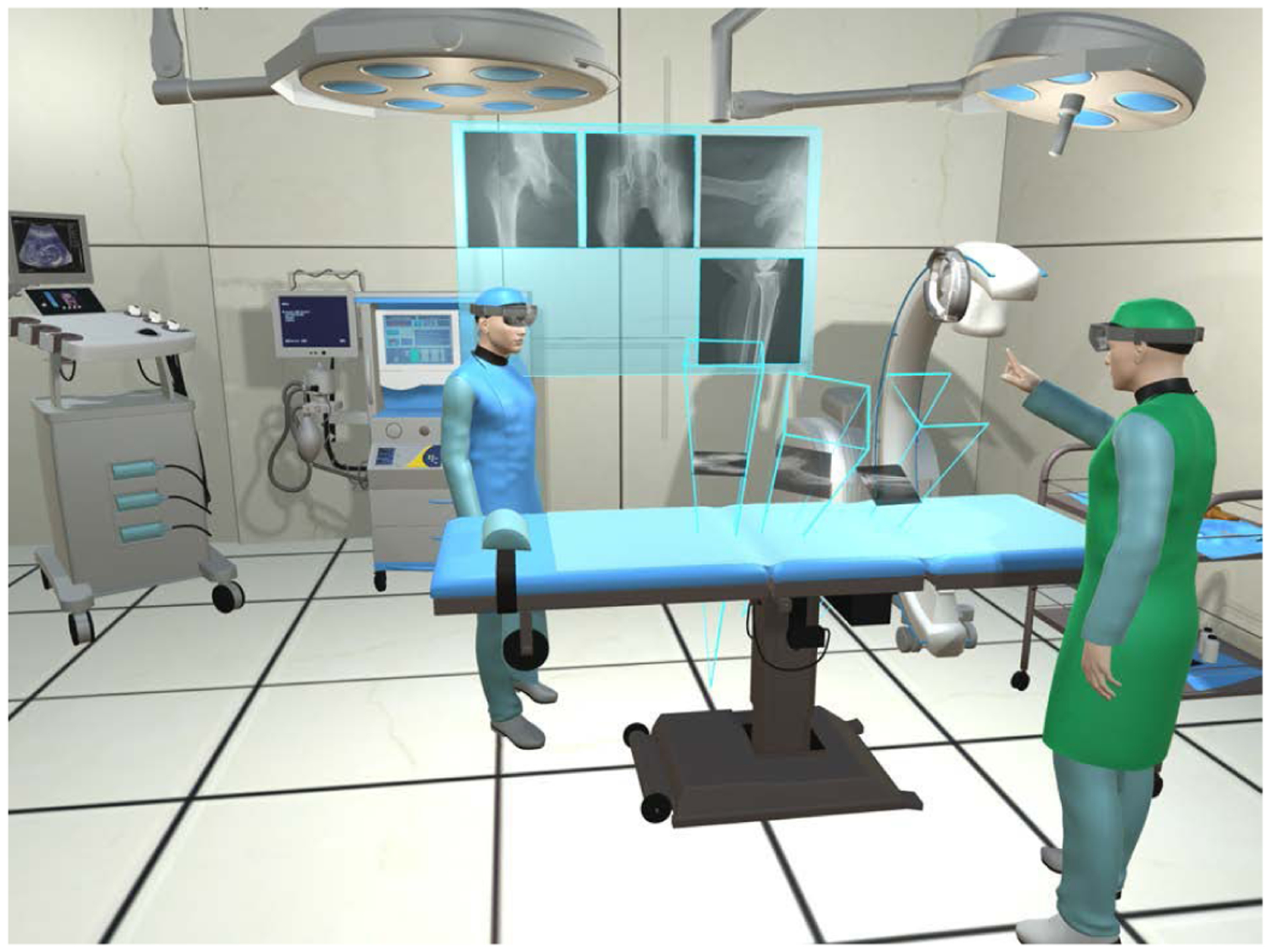
All acquisitions can be documented and later reviewed with all their corresponding spatial and temporal information. Spatiotemporal-aware AR allows the trainees to watch the surgery’s progress and revisit the actions taken based upon each image.

Our proposed AR landscape is enabled by exploiting all involved frustums to move spatial information between different bodies, hence allowing multiple users to connect simultaneously. In Fig. 8, we show hypothetical procedures and further demonstrate that the scanner, crew and the technician can all share this common AR experience through HMDs, thus jointly benefiting from the augmented procedure. As highlighted in the figure, our system relies on 2D C-arm fluoroscopy, thus the standard workflow is only minimally altered. The surgeon can always alternate between fluoroscopy-based guidance and the AR view to ensure safe drilling. The figure also signifies the advantage that the surgeon does not need to take his/her gaze away from the patient site during implant placement. As shown in Fig. 9, all the spatial and temporal information can be documented for post-operative review and training.

## III. Experimental Results

### A. System Setup

Our system comprises an ARCADIS Orbic 3D C-arm (Siemens Healthineers, Forchheim, Germany) as an intra-operative X-ray device that automatically computes the cumulative area dose for each session. The immersive AR solution was built using the Unity cross-platform game engine (Unity Technologies, San Francisco, CA, US) and was deployed to an optical see-through HMD, the Microsoft HoloLens (Microsoft, Redmond, WA). To jointly co-localize the augmented surgeon and the C-arm scanner, a second HoloLens device with inside-out SLAM capabilities was attached near the X-ray detector. The two HMDs shared their spatial anchor, a rich feature reference region in the common environment, over a wireless local network, allowing them to remain synchronized and establish spatial awareness. This connection was enabled through a TCP-based sharing service running on an Alienware (Dell, Round Rock, TX, US) laptop server with an Intel i7-7700HQ CPU, NVIDIA GTX 1070 graphics card, 16 GB RAM, and Windows 10 operating system. The 16 bit 1024 × 1024 single channel X-ray images from C-arm were transmitted to the server computer over a direct Ethernet connection, and then converted to 8 bit grayscale while keeping the original resolution in order to display them properly on the HMD as well as to reduce the data size before uploading them to the HMD. The frame per second update for the HMD device was 60, and the display resolution was 720 p.

To minimize the chance of tracking failure, we mounted the tracking HMD on the C-arm such that the integrated vision cameras on the HMD observe the static structures such as walls and ceiling. Consequently, the chances of blocking the tracking cameras on the HMD during the procedure are diminished.

### B. System Calibration

**Fig. 10.**
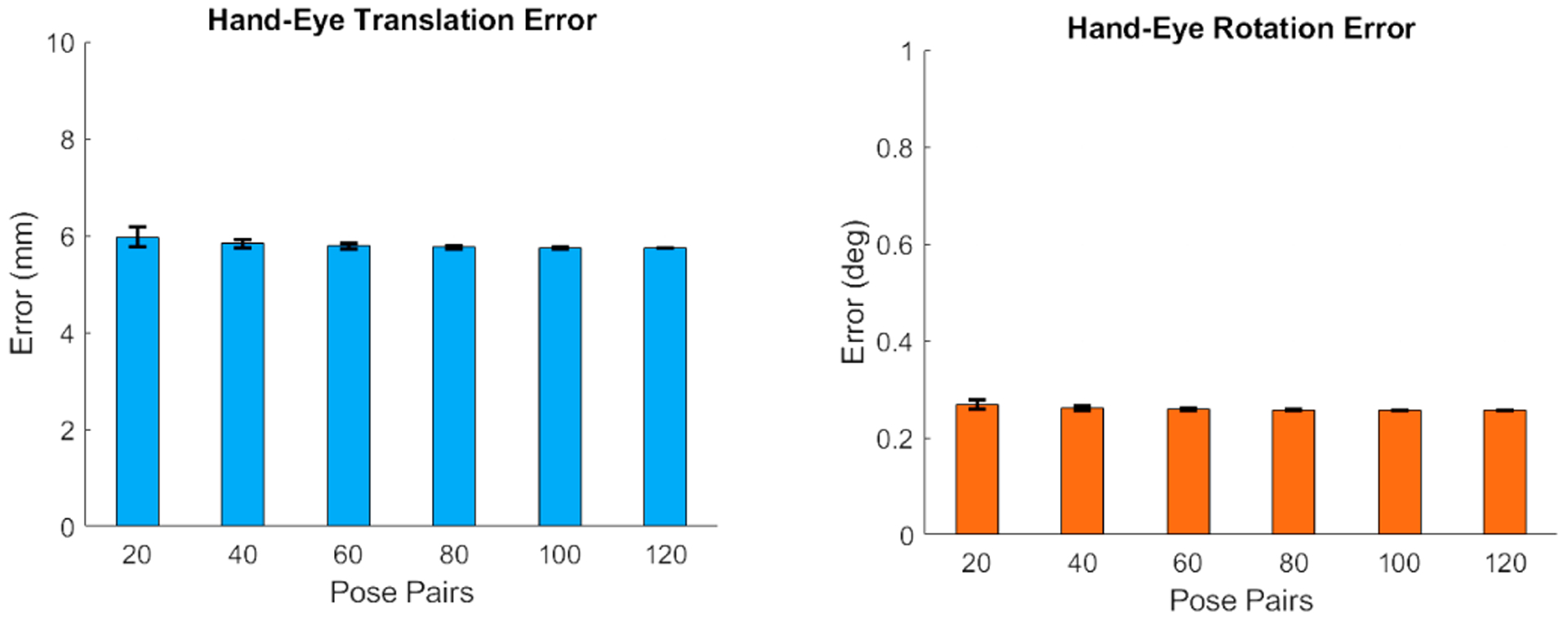
Mean and standard deviation of the translational and rotational errors for the hand eye calibration step are shown in the left and right plots, respectively. For each *N* number of pose pairs shown on the horizontal axis (except the last column which considers all the available data), the experiments were repeated 100 times by each time sampling *N* poses from the total 120 available poses and computing the hand-eye calibration.

To solve the hand-eye calibration problem in Eq. 3, 120 different pairs of corresponding poses were recorded from the visual tracker on the C-arm as well as an external infrared tracking system that tracked the C-arm source. Fig. 10 presents the error for this offline calibration step given different sampling for the pose pairs.

The localization quality of the SLAM-based visual tracking system on the scanner, *i*.*e*. the HMD on the scanner, was compared to a ground-truth provided by an external tracker. We measured rotational errors of (0.71°, 0.11°, 0.74°) with a norm of 0.75° and translational errors of (4.0, 5.0, 4.8) mm with a norm of 8.0 mm along the (*X, Y, Z*)-axes, respectively [41].

### C. Experiments

Eight orthopedic surgeons and residents from Johns Hopkins Hospital participated in pre-clinical user studies and performed two surgically relevant tasks while utilizing interactive flying frustums in an immersive AR environment. The surgeons are referenced as **Pi**. **P6** is an attending surgeon. At the time of the study, **P7** and **P4** were in their final year of residency, **P5** in their fourth year, **P3** in their third year, **P2** in their second year, and **P1** and **P8** were first-year residents.

**Fig. 11.**
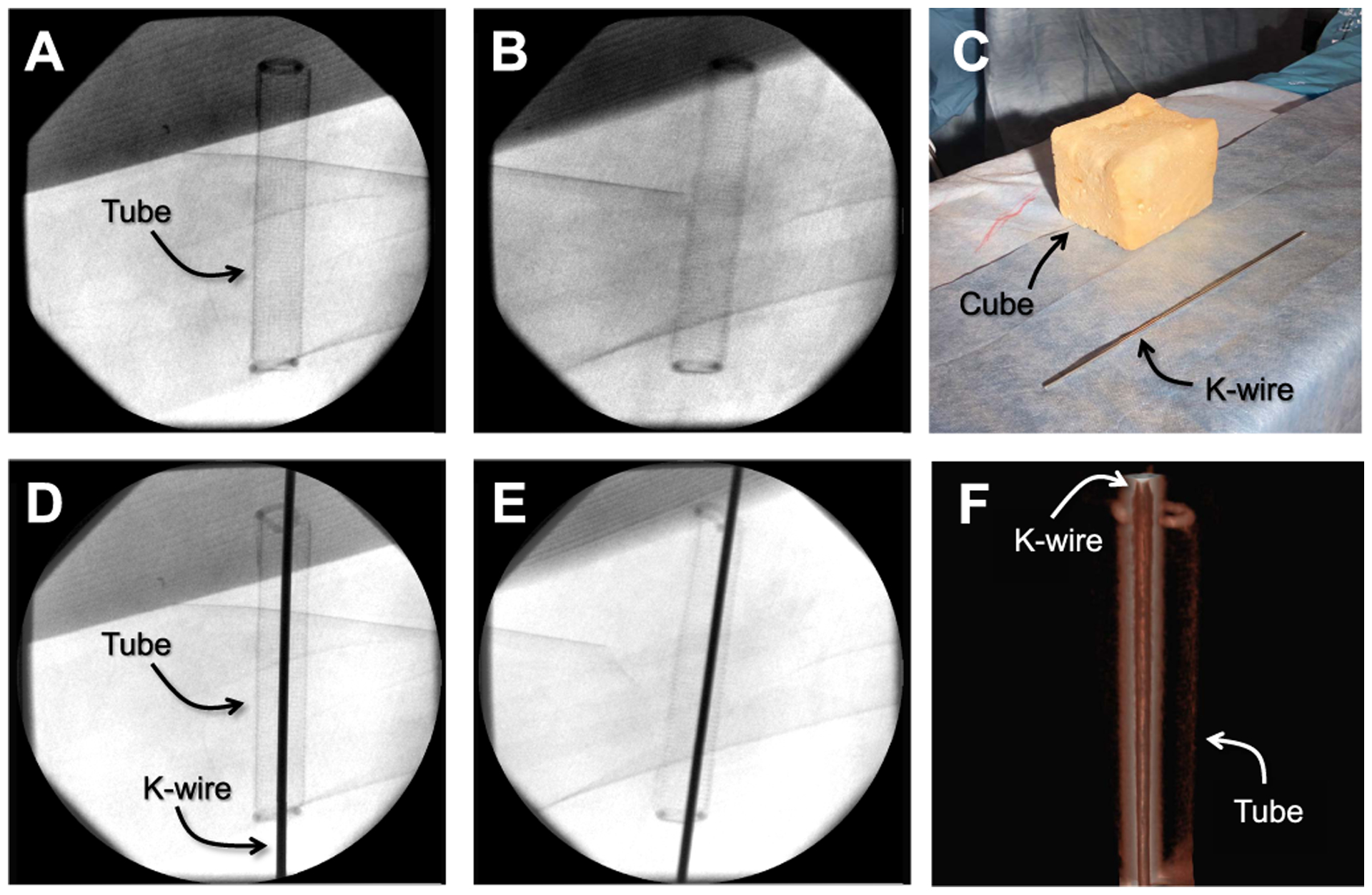
**A-B** are the X-ray images of the cubic phantom shown in **C**. In **D-E**, the X-ray images of the same phantom are shown after a K-wire was successfully inserted inside the tube. **F** is the CBCT scan of the phantom which was acquired for verification. Due to metal artifacts, the tube does not exhibit strong contrast.

In the first procedure, we focused on the correct placement of a K-wire to repair complex fractures. To emulate the K-wire placement through the superior pubic ramus (acetabulum arc), we used radiopaque cubic phantoms, as seen in Fig. 11–C. For direct comparison, we used the same setup that was used by Fischer *et al*. [22]. Each cube consisted of a stiff, lightweight, and non radiopaque methylene bisphenyl diisocyanate (MDI) foam. Since the superior pubic ramus is a tubular bone with a diameter of approximately 10 mm, we used a thin aluminium mesh filled with MDI that was placed inside each cube and served as the bone phantom. The two ends of the tubular structures were complemented with a rubber radiopaque ring. Each subject was asked to place a K-wire with a diameter of 2.8 mm through the tubular phantom using a surgical drill (Stryker Corporation, Kalamazoo, MI, US).

**Fig. 12.**
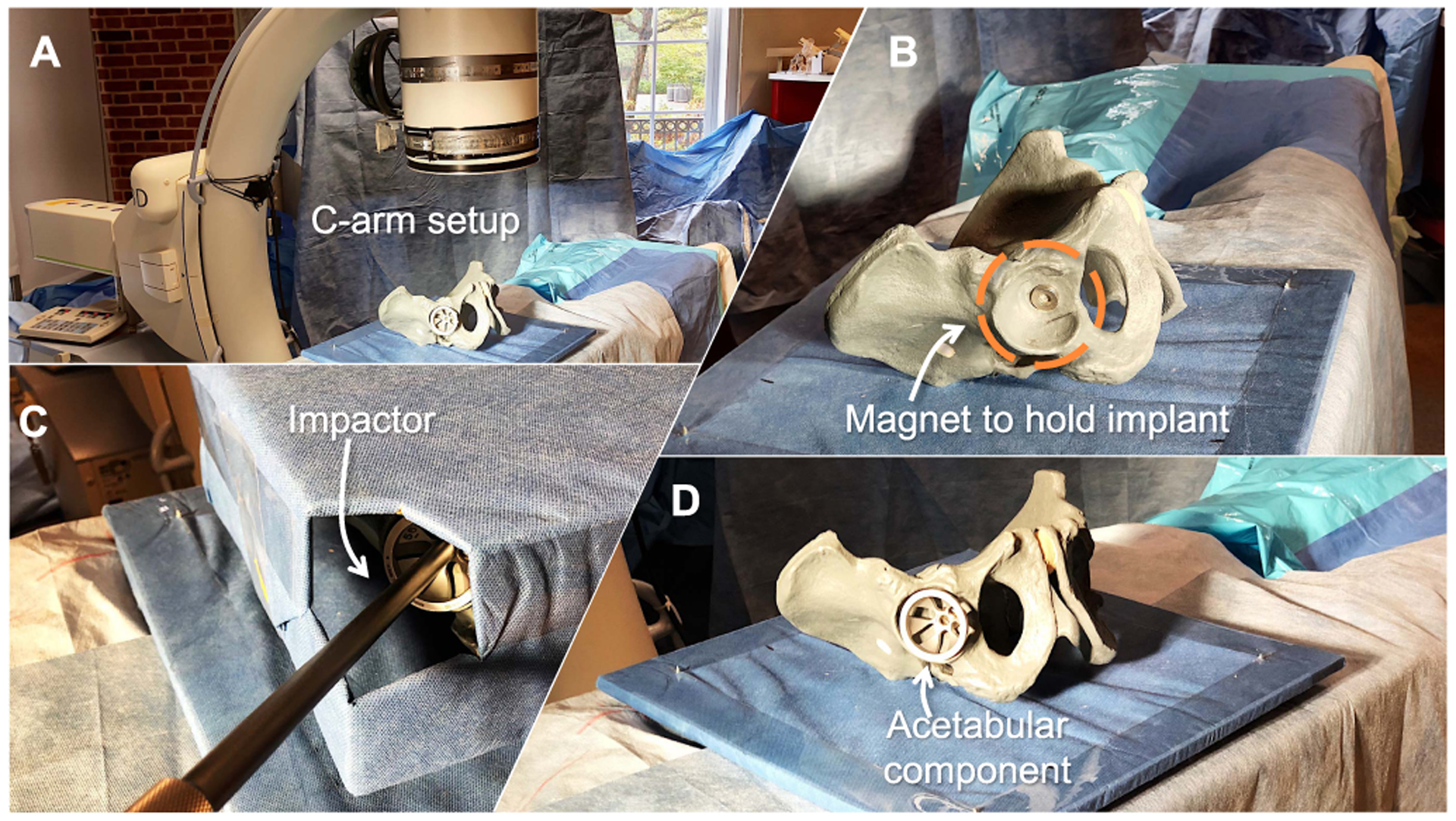
In **A** the setup of the C-arm, pelvic phantom, and the acetabular cup are shown. **B** is a close-up view of the phantom with an empty acetabular socket and a magnet for holding the implant in position. Image **C** shows the impactor while it is placed by a surgeon during the experiment, and **D** shows the successfully placed cup in the acetabulum.

For the second procedure, we constructed a total hip arthroplasty mock setup by using a radiopaque pelvis phantom with a magnetic acetabulum to fixate the acetabular cup (Fig. 12). For direct comparison, we adopted the same experimental setup that was suggested by Alexander *et al*. [44]. The cup was attached to a straight cylindrical acetabular trialing impactor (Smith & Nephew, London, UK) allowing the operator to guide the cup. Since the ideal orientation of the implant is unknown, we use abduction and anteversion angles that lie in a safe zone defined by landmarks on the pelvis as described in [45].

Initially, each surgeon received a brief introduction to the Microsoft Hololens, preparing them to properly mount and use the HMD. To further instruct them on our AR application, pre-recorded training X-ray images were loaded onto their HMD, allowing them to become familiar with the interface, planning procedure, and the interaction mechanism using hand gestures.

After the required planning images were acquired by the proctors, each surgeon planned their respective procedure in AR and performed the drilling task into the cube or placed the acetabular component into the pelvis. During the procedure, they were explicitly allowed to order as many X-ray shots from any perspective that they considered necessary.

We recorded the planing time, the time it took them to execute the procedure, number of fluoroscopic acquisitions, and the cumulative radiation dose as it was measured by the scanner. Finally, for the verification and accuracy measurement, we acquired a 3D cone-beam CT (CBCT) scan of the phantoms with their respective implants. It is important to note that distance is not defined for two non-parallel lines. The distance we computed from the drilled path to the desired path, i.e., the average distance from the wire to the center-line of the target structure, is consistent with the past literature [22], and provides an intuition regarding the range of error.

### D. Results

Tables I and II comprise the performance of every participant in the experiments. Table I contains the measurements for the K-wire insertion, and Table II presents the procedural outcome for the acetabular cup placement. We separate the interventional time measurements into *i*) *planning time*, the time it took each surgeon to plan their procedure in AR, and *ii*) *execution time*, determining the duration of the insertion/placement of the instruments. Furthermore, we recorded the number of X-ray acquisitions and the respective dose for each user. Finally, to assess the overall performances, we computed the average distance of the K-wire from the center of the tube at the entry and the exit surface of the tubular structure, and the abduction and anteversion angles of the acetabular implant, based on standard guidelines. Table III shows the results from the baseline standard operating procedure (SOP) using conventional fluoroscopic guidance.

**Fig. 13.**
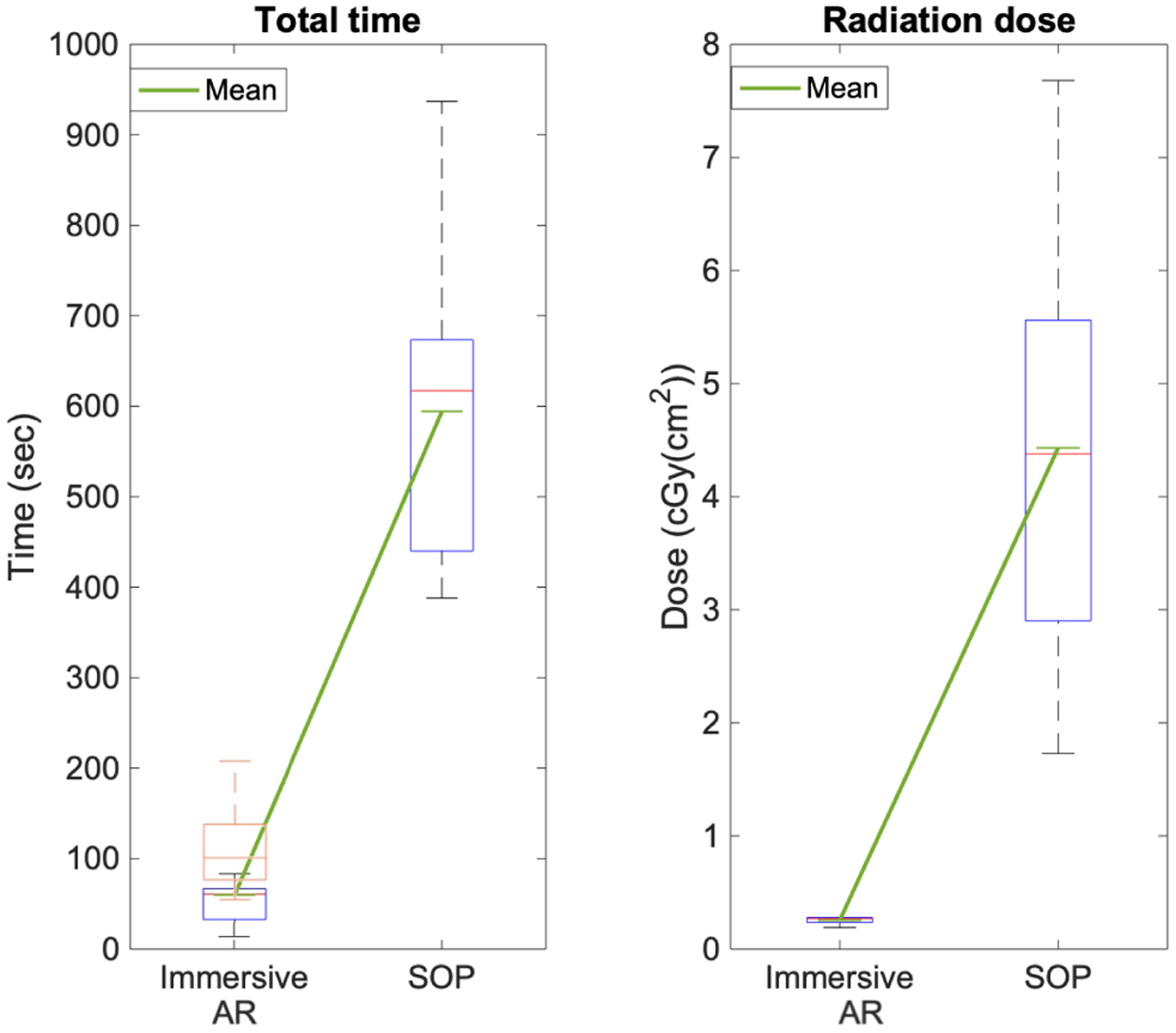
The plots present the execution time and total radiation dose during K-wire insertion using the AR supported approach and SOP. On the leftmost plot, the blue boxplot is the execution time with AR, whereas the orange boxplot is the total time including the planning phase. The green lines show the mean values for each of the groups.

Table IV provides a comparison of the mean and standard deviation (SD) values of the K-wire insertion errors using our immersive AR system with a previous non-immersive AR system [22] as well as the SOP. Combining the planning and execution times, the AR procedure took on average 111.25 sec versus the 594.3 sec during SOP. Fig. 13 depicts this comparison. On average, the surgeons used 2 fluoroscopic shots with a combined dose of 0.255 cGY(cm^2^) per user and committed an insertion error of 4.76 mm. Given the eight samples, the population mean for the drilling error, based on the 95% confidence intervals, is between [2.60 – 6.92] mm.

**Fig. 14.**
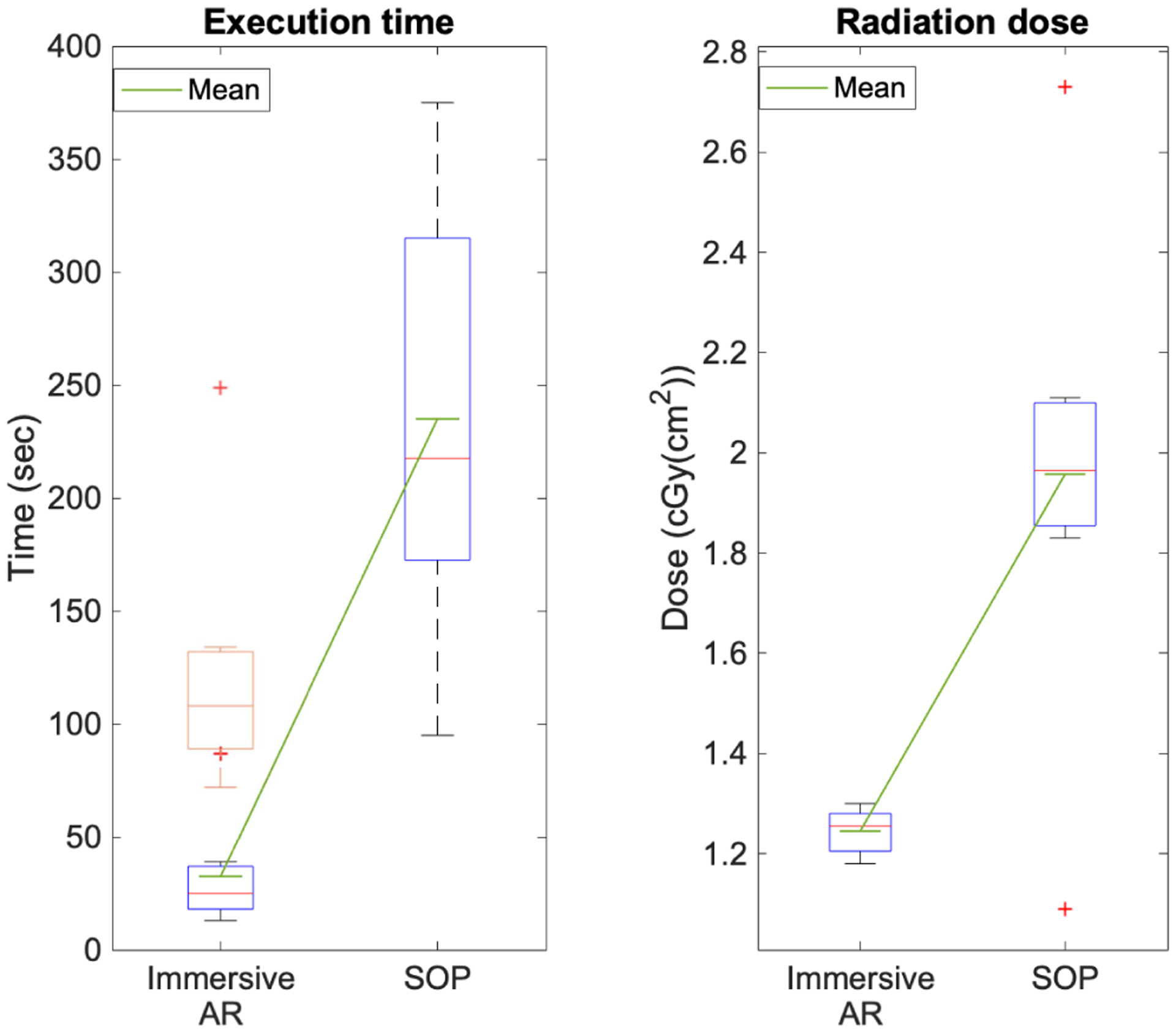
Comparison of time and total radiation dose during cup placement with AR and SOP approaches. The orange boxplot represents the total time including the planning time. The red (+) denote outliers, where in the leftmost plot the top sign belongs to the orange boxplot, and the bottom (+) to the blue plot.

**Fig. 15.**
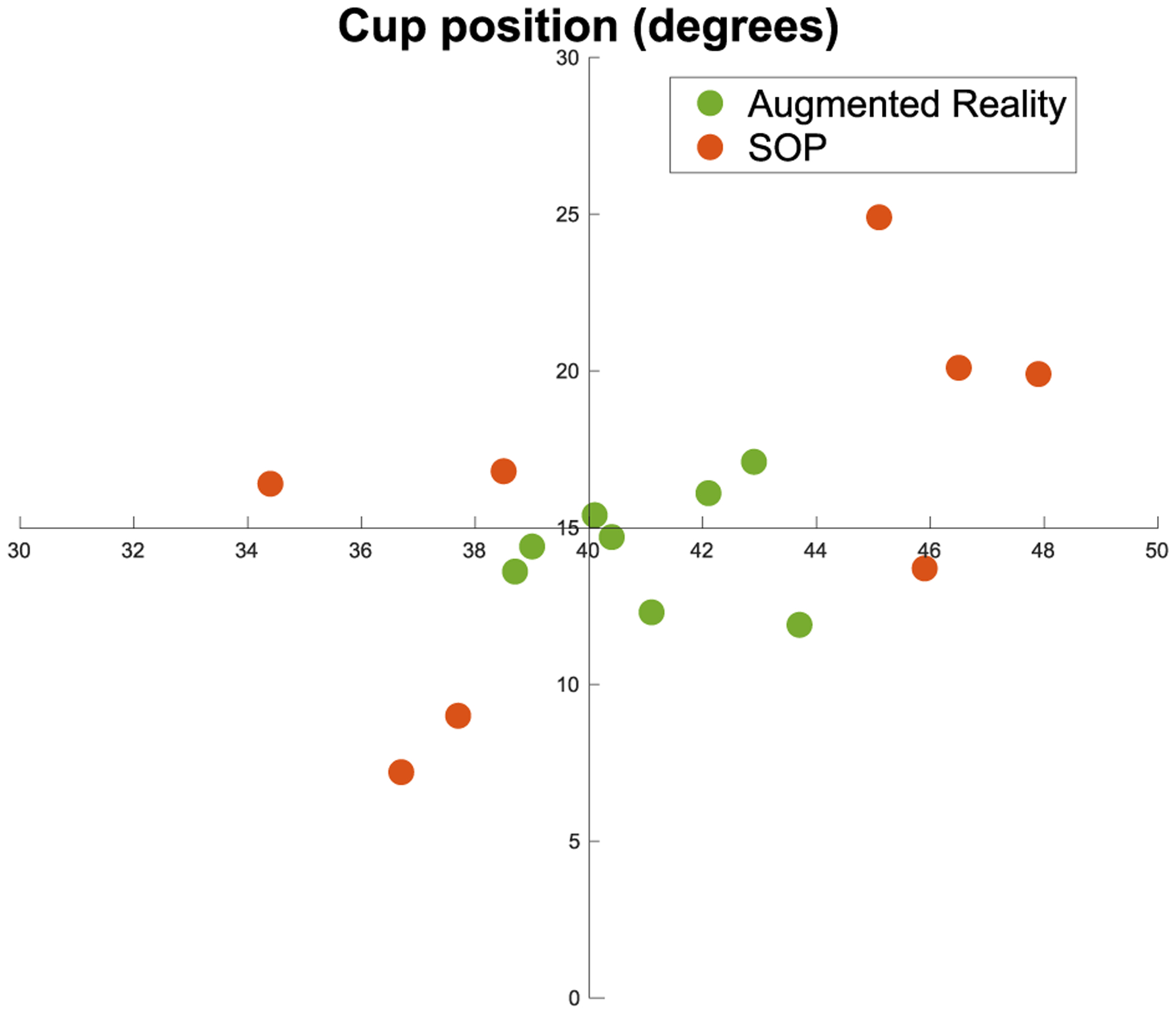
Anteversion and abduction angles are shown after acetabular cup placement using AR support and SOP. The horizontal axis represents the abduction angle, and the vertical axis represents the anteversion—the center of the plot corresponds to the desired angles of 40° and 15°. The farther data points from the center signify higher errors committed by the user. The AR method resulted in a stronger cluster near the center, while SOP yielded higher errors and more outliers.

Correspondingly, we present the outcome for the acetabular cup placement procedure with the immersive AR system in Table V, comparing it to a previous non-immersive AR application [44] and SOP. Using SOP, it took surgeons on average 235 sec to place the cup and under AR a combined time of 122.38 sec was achieved. For the AR setup, we acquired 8 X-ray images with an average dose of 1.25 cGY(cm^2^) per surgeon, whereas 14 fluoroscopic images with a dose of 1.96 cGY(cm^2^) were acquired during SOP. With the AR system, the average errors were 1.57° and 1.46° for the abduction and anteversion angles, respectively. Based on the 95% confidence interval, the mean error for abduction and anteversion are between [0.72° – 2.44°] and [0.72° – 2.20°], respectively. Under SOP the respective angles were 4.76° and 4.78°. Figs. 14 and 15 present the outcome with respect to time, radiation dose, and individual rotational measures for the acetabular cup placement experiments using AR and SOP. The immersive AR results show an SD of respectively 89.88 sec and 32.5 sec for planning and execution time, 0 for the number of X-ray images, 1.25 for the dose, 1.24 for the abduction error and 1.07 in the anteversion error. Comparable to Tables I and II, Table III displays the individual participants performance during each of the two SOP. To statistically evaluate our findings, a two sample t-test was performed and the results are reported in Table VI. The threshold for statistical significance is considered as *p* = 0.05. We tested the results of our immersive AR system against the results from the NI-AR system and SOP for both the K-wire insertion procedure and the acetabular cup placement. We did not test the number of acquired X-rays, since in these experiments they turned out to be a constant value for each procedure.

## IV. Discussion

We evaluated our spatially-aware AR system in two clinically relevant procedures, *i*) the placement of K-wires through tubular structures for fracture repair tasks, and *ii*) placement of acetabular components into the hip socket for total hip arthroplasty. We selected these two high volume procedures among the many other applications which can be enabled by our interactive AR system, as they each represent a class of common orientational alignment and localization tasks that are prevalent across different fields of image-guided surgery.

For the K-wire insertion procedure, the immersive AR system performed significantly faster than the conventional SOP, yielding less than a fifth of the time (Fig. 13). Table IV demonstrates a detailed comparison of our system, not only with the SOP as an established baseline, but also with a previously presented non-immersive mixed reality method based on RGBD sensing and intra-operative CBCT imaging [22].

With the AR system every surgeon used exactly 2 X-ray images, which were the 2 images required for procedure planning. Despite explicitly allowing them to take as many radiographs as they desire, no one of the surgeons requested additional X-ray images. As mentioned above, during SOP, surgeons inserted the K-wires with an average number of 40.86 fluoroscopic images and with an average dose of 4.43 cGY(cm^2^), compared to the (statistically) significantly lower dose of 0.255 cGY(cm^2^), which was emitted during the AR procedures. The RGBD-CBCT system in [22], [23] yielded on average 2.14 X-rays, although it required a pre-procedural CBCT scan of the phantom, inducing the higher radiation dose of 1.6 cGY(cm^2^).

Finally, evaluating the outcome of the procedure with regard to the drilling error, AR (4.76 mm) outperforms RGBD-CBCT (5.13 mm), both being marginally worse than SOP (4.61 mm). The differences in accuracy were not statistically significant. Considering that we only instructed the surgeons to drill through the tube and not precisely through the center of the tube, we regard these difference as negligible. It is important to note that, our AR system performed similar to the conventional X-ray method in terms of accuracy, while reducing time by a factor of 5, number of fluoroscopic acquisitions by a factor of 20, and the radiation dose by a factor of 17.

We observed that, in addition to the planning information, the surgeons took multiple other considerations into account while deciding on the insertion path. One of which was direct visualization of the X-ray images that they acquired for planning. Observing the C-arm pose with respect to the patient, and the visualization of the images within their viewing frustum, assisted them in better localizing the target structure. In our setup, tactile feedback did not play a significant role; however, in reducing real fractures, haptic feedback further assists the surgeon in identifying whether the wire is inserted in specific anatomy or not.

The SLAM-based error for HMD is dominantly along the principal axis of the viewer, i.e., direction pointing away from the user to the scene. When the users are presented with a trajectory to follow, we observed that they naturally place their heads in the direction of the target trajectory for optimal visualization and ease of alignment (Fig. 3). This configuration is advantageous since the maximum tracking uncertainty is in the direction of the drilling trajectory, which is the direction that is not relevant to the drilling task. This is because the amount of penetration of the drill is easily identified from X-ray images. This uncertainty behaviour in AR has been previously investigated by Hoff *et al*. [46].

A similar trend to the K-wire experiment is observed with the measurements for the placement of the acetabular cup, demonstrating the effectiveness of our AR system, we compare it against SOP and a NI-AR system as presented in [44]. As shown in Fig. 14, the execution time is considerably lower using AR; even when combining planning and execution time, it took the surgeons 122.38 sec, which is nearly half of the 235 sec that they needed under SOP and comparable to the 110.6 sec with NI-AR. Both differences are statistically significant. Furthermore, the number of fluoroscopic images were reduced; every surgeon used exactly 8 images, which are again merely the images required for planning. This resulted in an average dose of 1.25 cGY(cm^2^), which is significantly lower than with SOP, where the surgeons used an average of 13.75 radiographs with an average dose of 1.96 cGY(cm^2^), and lower than with NI-AR where one pre-procedure CBCT lead to a dose of 1.83 cGY(cm^2^). The objective of this procedure was to achieve abduction and anteversion angles of 40° and 15°, respectively, which lie in the clinical safe-zone [45]. The respective errors are shown in Table V and Fig. 15. The outcome distinctly displays a more accurate cup placement using the spatially-aware immersive AR system (1.57° & 1.46°) compared to the SOP (4.76° & 4.78°), compared to the NI-AR system (1.78° & 1.43°) the abduction error is slightly less, whereas the anteversion error is marginally higher (0.03°). The differences in the abduction and anteversion errors between the immersive AR system and SOP are both statistically significant.

Concerning the planning and execution time, some participants such as **P2**, **P6**, and **P8**, who had previous experience with AR HMDs and were familiar with our system performed faster than other users, especially compared to **P1** and **P7**, who had no prior exposure to HMDs. In order to reduce the disparity in acquaintance with the technology, each participant completed a short training in which they were familiarized with the headset and interaction techniques used in our software. Despite the same training session, the differences mentioned above still seem related to experience with AR. We expect the performances to level after users gain more experience with the system. These outcomes are not surprising as any new technology requires time and experience to exploit its full potential.

In almost all orthopedic procedures, we rarely use live fluoroscopy and rely almost exclusively on still images. Therefore, the frame-rate for these procedures appeared entirely adequate. The resolution also seemed satisfactory for the experiments that we performed. No users complained about not being able to *“see the planning annotations”*. However, if the system wanted to be adapted to other fields, such as interventional neuroradiology — the suitability of resolution, contrast, brightness, frame-rate, etc. may need to be further investigated.

For both procedures the deployment of our AR system lead to a comparable or higher accuracy, fewer X-ray images with a consequently lower radiation dose. For the total time, it has to be noted that our planning time does not include the recording of the X-ray images that were necessary to plan the procedure. This step however can be fully automated, resulting in an immediate availability of the fluoroscopic images.

## V. Conclusion and Future Work

We presented the embodiment of a novel interaction concept based on spatiotemporal-aware AR. In our work, we aimed to provide meaningful registration and visualization without the need for tracking patients or tools with outside-in navigation hardware. Instead, we brought intuition to visualization by connecting the viewing frustums of the scanner with the surgical team. For the two orthopedic use cases presented in this manuscript, our immersive AR system demonstrated improvements in time, number of X-ray acquisitions, radiation dose, and outcome during cup placement.

The most significant source of error is from the localization of the AR HMD using SLAM. This error is present both during calibration and application. If additional sensors such as IMUs and depth cameras would constraint the tracking algorithm in the future, we can expect improved tracking and smaller drifts.

As computer vision methodologies enter the medical and AR domains, the accuracy and robustness of medical AR systems will also increase. This work presents a general end-to-end workflow for integration of AR into orthopedic surgery, which will also observe dramatic improvement as such progress occurs.

**Fig. 16.**
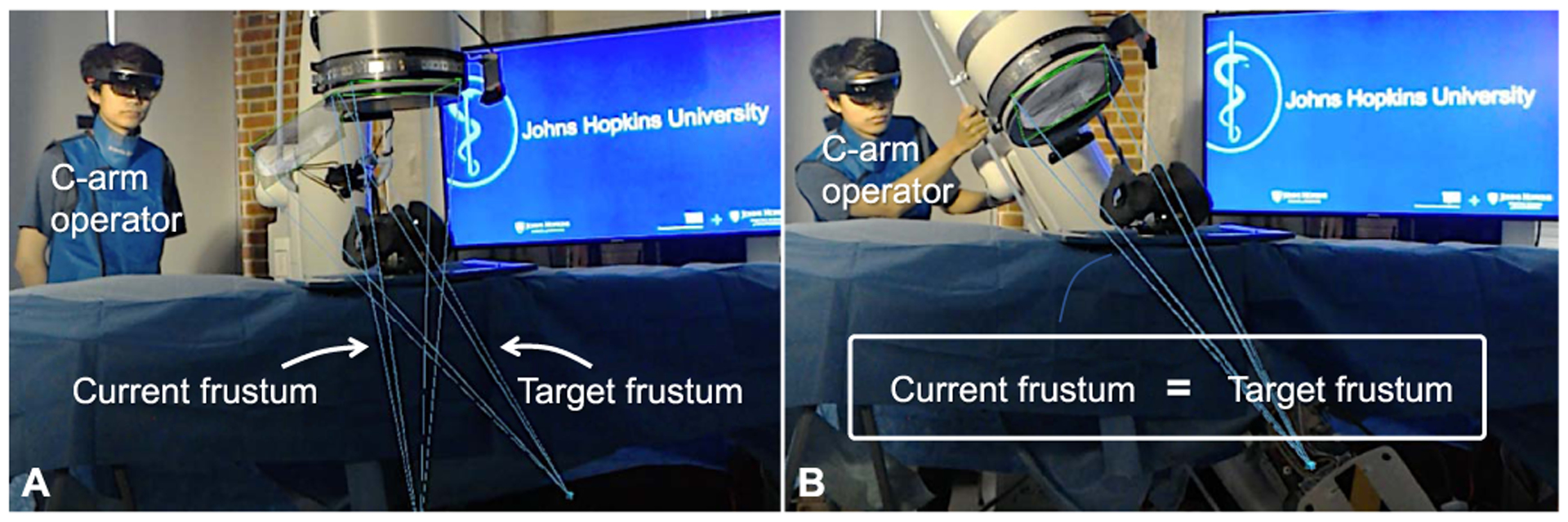
Visualization of a target frustum (**A**) allows the C-arm operator to align the current C-arm frustum with the surgeon’s desired perspective (**B**) and eliminate the waste of time and radiation during fluoro hunting. This concept is an example of the capabilities of interactive frustums on moving information between different stake holders in the OR, *i.e.* surgeon, patient, X-ray technician, staff, etc.

The spatiotemporal awareness inherent in AR overhauls the ill-posed communication between the surgeon, staff, and information; *e*.*g*. Fig. 16 shows the potential role of flying frustums and AR in effectively communicating desired X-ray views to the technician, eliminating unfavorable views and reducing the staff burnout.

A significant advantage of our system is that it is based on 2D C-arm fluoroscopy —- and therefore does not diverge from the standard workflow. The standard C-arm is always present in the OR to take confirmatory images. Consequently, the surgeons are not required to solely rely on the AR system during a procedure. Rather, its value lies in guiding the surgeon to a narrow area of interest and more importantly, to align them with the right trajectory. The surgeon can then seamlessly switch between standard fluoroscopic images and the AR view to guarantee accurate drilling. We believe this is useful, translatable, safe, and novel.

An application of AR outside the OR is *“surgical replay”*, where the residents can review the surgery, accompanied with its temporal and spatial information including all the X-ray acquisitions and optical point-clouds from the patient site. This enables the medical trainees to identify distinct actions that were taken by the experienced surgeon based upon each image. Access to such 3D post-operative analysis has the potential to dramatically improve the quality of surgical education.

In a training environment, such as in most academic centers with residents and fellows — this ability for all users to wear the HMD and view the same annotations and AR guidance would be helpful. The more experienced user could ensure that the anatomy had been annotated appropriately and that the AR guidance trajectories are at the correct location.

Direct visualization of X-ray images within their corresponding viewing frustums delivers intuition that effectively unites the content of the 2D image with the 3D imaged anatomy. In this setting, images from various perspectives can be grouped within their frustums to form multi- or extended-view representations of the anatomy. The interlocked frustums shown in Fig. 18 are examples for such visualization concept, that can particularly benefit interventions where leg-length discrepancies or malrotations in tibio- and lateral/distal-femoral angles are major concerns.

**Fig. 17.**
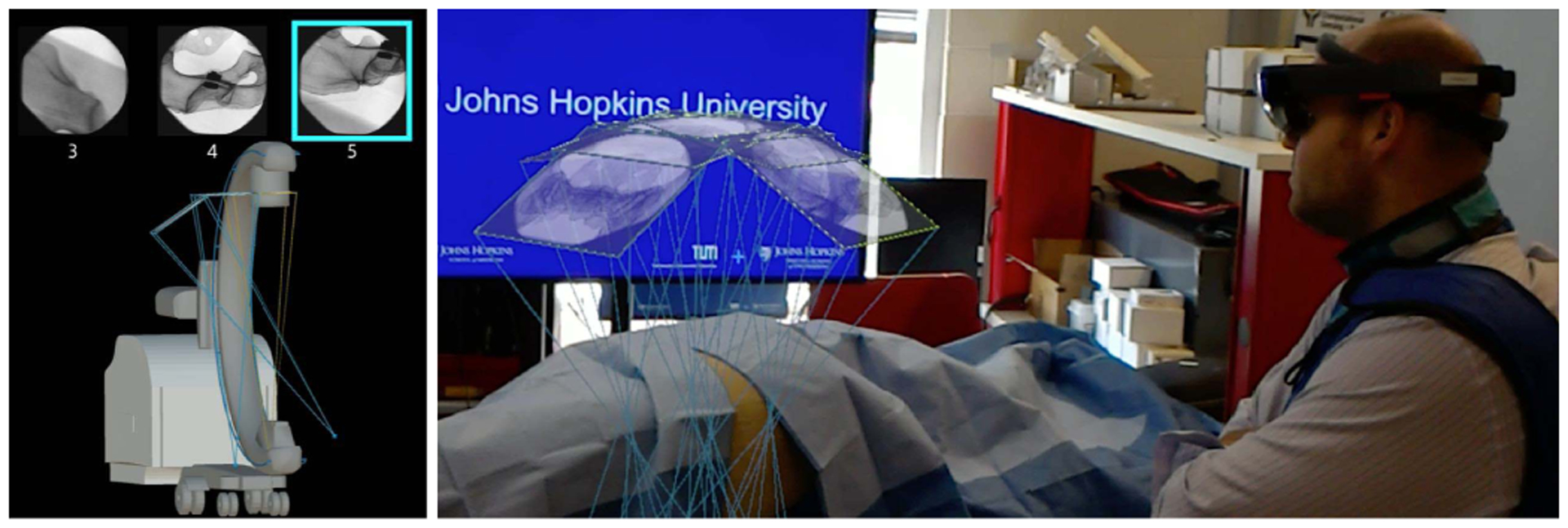
Spatial and temporal information from the surgery can be recorded and reviewed after surgery. On the left side an interface is shown allowing the surgeon to select images, which he can then observe geometrically accurate in space, as shown on the right side.

**Fig. 18.**
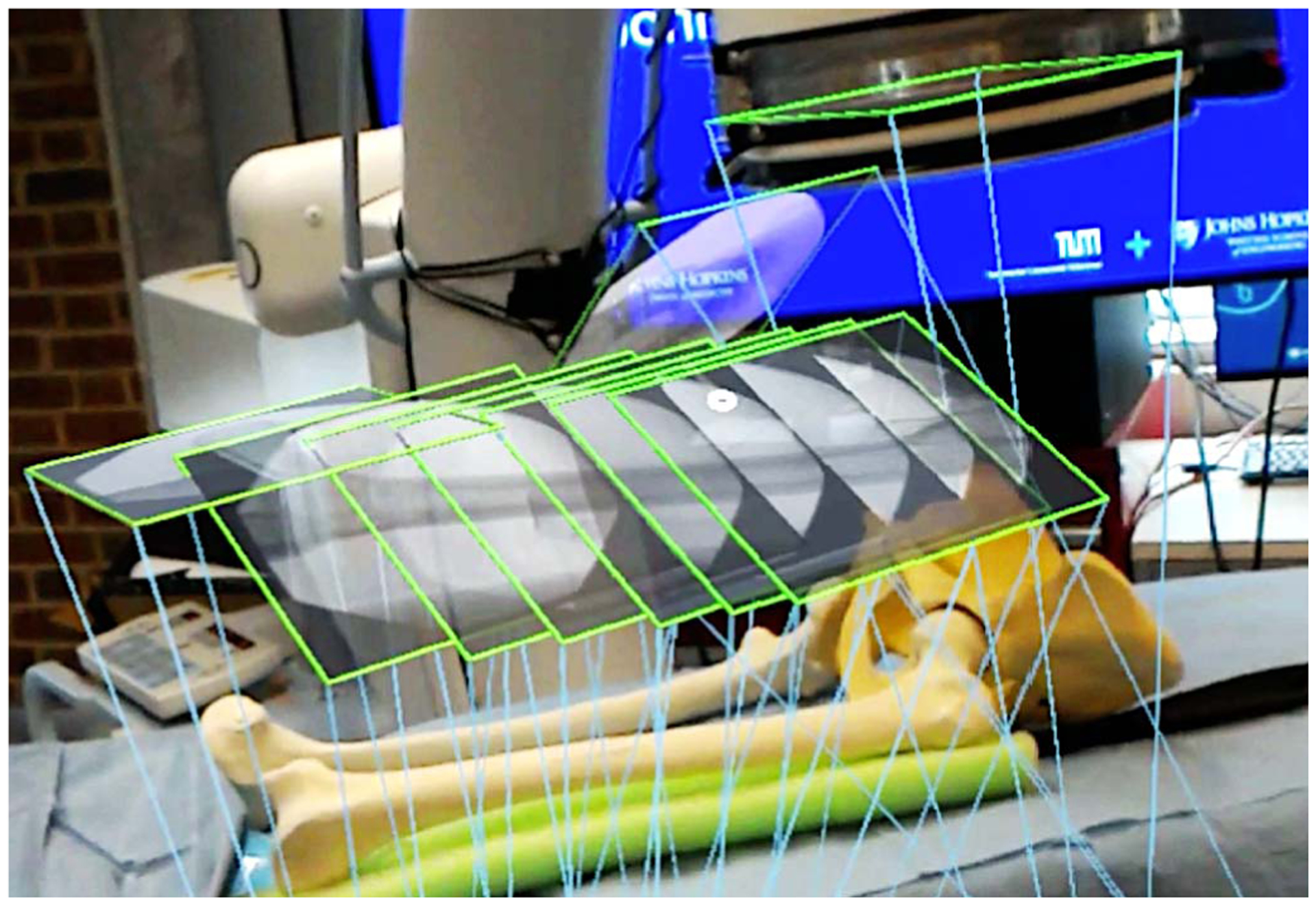
The interlocking of multiple X-ray frustums enables visualization of large anatomical structures. In this figure, multiple images are acquired on a co-linear trajectory and are locked to each other to form a quasi-panoramic view of the bone.

In this work, we demonstrated that two elements share the AR experience; the first one was the augmented surgeon, and the second one was the imaging observer which was the C-arm scanner that used a calibrated HMD attached to it. With the same design, any number of other users can connect to the same AR session, collaborate, and share an experience. Though assessment of each of these concepts presented in Figs. 16, 17, and 18 requires an additional approved study, we believe their introduction to the community paves the way in opening new paths for research in this area and expedites the translation of AR-based solutions into future ORs.

Despite that our solution delivers spatial awareness, it should not be regarded as a surgical navigation system. This is because marker-less tracking, currently, cannot deliver the level of accuracy achieved by marker-based surgical navigation or robotic systems. It is important to note that, as most AR systems display the data anchored to the surrounding environment, tracking with respect to the environment has become an inherent component in modern AR systems. In an optical see-through HMD, the integrated camera sensors are used to run SLAM in the background, construct a dynamic map from the environment, and localize the HMD within that environment. Despite that this marker-less mapping and localization cannot deliver the same level of accuracy as the external navigation systems with explicit tracking nature, it delivers spatial awareness for the visualization of information. Hence, we regard our solution as a spatially-aware advanced visualization platform that enhances the interaction across the surgical ecosystem and promotes effective collaboration and team approach.

The widespread adoption of human-centerd AR in interventional routines requires careful considerations regarding surgeons’ experience. The interaction with the virtual content using intuitive hand gestures, and resolving the perceptual ambiguities between real and virtual that occur due to vergence-accommodation conflict, can greatly contribute to the acceptance of AR technologies. Furthermore, development of artificial intelligence strategies can *i*) create semantic understanding from the surgical environment and augment surgeon’s intelligence, and *ii*) enhance the spatial mapping and co-localization, thus improving the stability of marker-less AR systems. Lastly, future integration of eye tracking systems into HMDs can circumvent the internal eye-to-display calibration, adjust the rendering based on each user, and replace unnecessary gestures with gaze-based interaction.

Regarding sterilization, since the HMD on the user’s head does not enter the operating field, it could be viewed in a similar way to surgical loupes, surgical hoods, headlamps — all of which are used daily and do not need to be sterilized.

Our solution that promotes the team approach, also demands the entire crew to get trained appropriately and be able to interact with the system comfortably. Therefore, time, money, and effort need to be spent to prepare the surgical team for such a digital transformation. From a hardware standpoint, other than HMDs, no additional hardware is required in the OR. Therefore, the device cost is lower than complex navigation-based or robotic solutions.

As shown in this paper, the introduction of new technology requires a user-centric design for its full integration into the clinical workflow. The ultimate goal may be the discovery of new surgical workflows enabled by the introduction of novel technology. This goal could only be achieved with a close and extended partnership between surgeons and technical experts, as well as the full integration of the advanced technology in the broad spectrum of surgical teaching, training, planning, workflow, and documentation.

## Figures and Tables

**TABLE I:** Outcome From the K-Wire Insertion Using Our Immersive AR System. Individual Performances Are Listed in Columns pi. Corresponding Mean and SD Values Can Be Found in Table IV. the Last Row Reports the Error That Was Measured Between the Inserted K-Wire and the Center of the Tube

K-wire	P1	P2	P3	P4	P5	P6	P7	P8
**Planning Time (sec)**	125	40	39	58	79	**22**	67	44
**Execution Time (sec)**	83	74	58	**46**	66	67	63	79
**# X-ray images**	2	2	2	2	2	2	2	2
**Dose (cGY(cm^2^))**	0.28	0.21	**0.19**	0.27	0.26	0.28	0.27	0.28
**Error (mm)**	8.23	5.71	9.02	3.26	6.94	**1.13**	1.59	2.23

**TABLE II:** Outcome From the Placement of the Acetabular Implant Using Our Immersive AR System. Individual Performances Are Listed in Columns pi. Corresponding Mean and SD Values Can Be Found in Table V

THA	P1	P2	P3	P4	P5	P6	P7	P8
**Planning Time (sec)**	162	70	117	88	64	**37**	71	110
**Execution Time (sec)**	87	39	**13**	19	17	35	26	24
**# X-ray images**	8	8	8	8	8	8	8	8
**Dose (cGY(cm^2^))**	1.27	1.3	1.23	1.26	**1.18**	1.25	**1.18**	1.29
**Abduction error (°)**	2.1	1	1.1	1.3	2.9	**0.1**	0.4	3.7
**Anteversion error (°)**	1.1	0.6	2.7	1.4	2.1	0.4	**0.3**	3.1

**TABLE III:** Results of The Respective SOP Presented in [22] and [44]. Columns Correspond to Individual Participants Performance. Corresponding Mean and SD Values Can Be Found in Table IV and Table V

	K-wire SOP						
	Q1	Q2	Q3	Q4	Q5	Q6	Q7
**Time (sec)**	937	686	617	464	636	388	432
**# X-ray images**	80	47	44	33	32	21	29
**Dose (cGY(cm^2^))**	7.68	1.73	3.54	4.38	5.62	2.69	5.38
**Error (mm)**	3.08	7.88	11.43	3.01	1.87	2.27	2.72

	THA SOP							
	R1	R2	R3	R4	R5	R6	R7	R8
**Time (sec)**	210	350	195	375	225	150	95	280
**# X-ray images**	13	15	15	16	13	13	6	19
**Dose (cGY(cm^2^))**	1.93	2	2.09	2.11	1.83	1.88	1.09	2.73
**Abduction error (°)**	7.9	5.9	5.1	6.5	5.6	2.3	1.5	3.3
**Anteversion error (°)**	4.9	1.3	9.9	5.1	1.4	6	1.8	7.8

**TABLE IV:** Mean and SD Values for K-Wire Insertion With the Immersive AR, NI-AR, and SOP. For Each Method Two Rows Show the Mean and SD Values, Respectively. For Immersive AR, the Time Is Separated Into First Planning and Then Execution

	Method	Time (sec)	# X-ray images	Dose (cGY(cm^2^))	Error (mm)
$\bar{X}$ *σ*	**AR**	**59.25 + 52** **(32.02, 24.23)**	**2** (0)	**0.255** **(0.04)**	4.76 (3.11)
$\bar{X}$ *σ*	**NI-AR**[22]	243.7 (84.00)	2.14 (0.69)	1.6 (0.17)	5.13 (**2.72**)
$\bar{X}$ *σ*	**SOP**	594.3 (188.0)	40.86 (19.38)	4.43 (2.00)	**4.61** (3.62)

**TABLE V:** Mean and SD Values for Acetabular Cup Placement With the Immersive AR, NI-AR, and SOP. For Each Method Two Rows Show the Mean and SD Values, Respectively. For Immersive AR, the Time Is Separated Into First Planning and Then Execution Time. In the #X-Ray Column of NI-AR, Only One X-Ray Is Denoted, This References the CBCT That Was Acquired Before the Experiment Which Is Reconstructed of 100 Digital Radiographs

	Method	Time (sec)	# X-ray images	Dose (cGY(cm^2^))	Abd. (°)	Ant. (°)
$\bar{X}$ *σ*	**AR**	**89.88 + 32.5** (38.85, 23.71)	8 (0)	**1.25** (1.25)	**1.57** (**1.24**)	1.46 (1.07)
$\bar{X}$ *σ*	**NI-AR**_[44]_	110.6 (**15**)	1 CBCT (0)	1.83 (**0.06**)	1.78 (1.37)	**1.43** (**0.66**)
$\bar{X}$ *σ*	**SOP**	235 (96)	13.75 (3.73)	1.96 (0.45)	4.76 (2.2)	4.78 (3.15)

**TABLE VI:** Results From Two Sample t-Tests of Our AR Method Compared to the Respective NI-AR Method and the SOP. The Upper Half of the Table Shows Results of the Statistical Evaluation of K-Wire Insertion With the AR and NI-AR Version as Well as AR and SOP. The Lower Half Contains the Corresponding Values for the Acetabular Cup Placement

	P-value	Time (sec)	Dose (cGY(cm^2^))	Error (mm)		Abd. (°)	Ant. (°)
K-wire	**AR / NI-AR**_[22]_ **AR / SOP**	3.27 · 10^−5^ 0.95· 10^−5^	1.25 · 10^−11^ 4.91 · 10^−5^	0.81 0.93		- -	- -
THA	**AR / NI-AR**_[44]_ **AR / SOP**	1.64 · 10^−6^ 0.12 · 10^−1^	0.34 5.39^−4^	- -	- -	0.49 0.31 · 10^−2^	0.22 0.14 · 10^−1^
